# Multiscale Simulations and Cryo-Electron Microscopy Reveal the Transition Pathway of Dengue Virus-like Particle Nanoassembly

**DOI:** 10.1021/acsnano.5c17047

**Published:** 2026-02-16

**Authors:** Venkata Raghuvamsi Palur, Guan-Wen Chen, Day-Yu Chao, Wen-Shuo Kuo, Ya-Na Wu, Jedhan U. Galula, Chun-Hsiung Wang, Fan-Chi Chen, Peter J. Bond, Jan K. Marzinek, Shang-Rung Wu

**Affiliations:** † School of Dentistry & Institute of Oral Medicine, College of Medicine, National Cheng Kung University, Tainan 70101, Taiwan; ‡ Bioinformatics Institute (BII), Agency for Science, Technology and Research (A*STAR), 30 Biopolis Street, #07-01 Matrix, Singapore 138671, Republic of Singapore; § Graduate Institute of Microbiology and Public Health, College of Veterinary Medicine, 200384 National Chung Hsing University, Taichung 40227, Taiwan; ∥ Department of Biological Sciences, National University of Singapore, Singapore 119077, Republic of Singapore; ⊥ Institute of Biological Chemistry, 71561 Academia Sinica, Taipei 11529, Taiwan; # Microbial Genomics PhD Program, National Chung Hsing University and Academia Sinica, Taichung 40227, Taiwan; ∇ Center for Allergy Immunology and Microbiome (AIM), China Medical University Children’s Hospital/China Medical University Hospital, China Medical University, Taichung 40447, Taiwan

**Keywords:** dengue virus-like particles, cryo-EM, CHO-K1 cell line, molecular dynamics simulations, maturation transition

## Abstract

Dengue virus (DENV) continues to impose a global health burden, and virus-like particles (VLPs) are promising vaccine candidates, owing to their ability to elicit broadly neutralizing antibodies. However, the lack of nanoscale structural insights into the VLP maturation process has limited rational engineering. Here, we report the cryo-electron microscopy (cryo-EM) structure of immature DENV serotype 2 VLPs, revealing prM–E spikes arrayed on a *T* = 1 shell, consistent with mature DENV-2 VLPs and distinct from the virion, which exhibits a *T* = 3 icosahedral lattice. To connect the experimentally determined immature and mature endpoint structures, we employed a multiscale molecular dynamics (MD) framework to assess sterically feasible transition pathways. The simulations support the steric feasibility of a sliding-rotating rearrangement in which trimeric prM–E spikes reorganize into flat E dimers (E–E) without clashes. In virions, trimers reorganize into extended rafts of three parallel E dimers, whereas in VLPs, which lack the long-range symmetry and geometric constraints of the *T* = 3 lattice, maturation proceeds via a less extensive rearrangement in which neighboring monomers form small triangular clusters of three dimers. In addition, the simulations reveal pronounced lipid core mobility during maturation, including transient and spatially localized lipid protrusion events that preferentially occur near regions undergoing protein rearrangement, consistent with a potential role for dynamic membrane remodeling in accommodating maturation-associated structural changes. We also established a stable Chinese hamster ovary (CHO-K1) producer cell line, enabling efficient production of immature DENV serotype 2 VLPs. Together, this work defines a structure-dynamics framework that links steric feasibility, membrane composition, and particle stability and outlines process-relevant, testable hypotheses to inform future engineering of dengue VLPs that may ultimately guide vaccine design.

## Graphical abstract

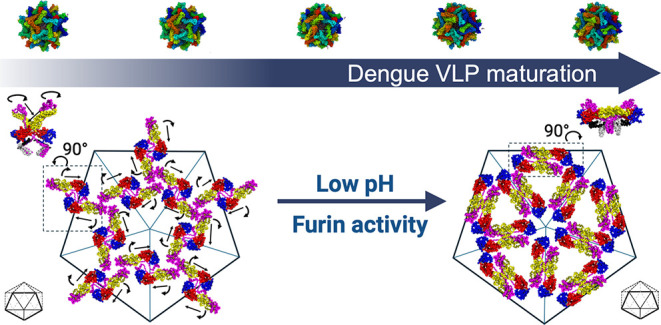

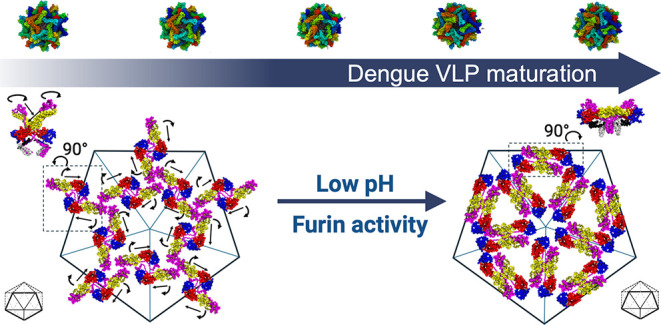

## Introduction

The global spread of dengue virus (DENV), a flavivirus primarily transmitted by *Aedes aegypti* and *Aedes albopictus* mosquitoes, leads to approximately 390 million infections annually.^1^ DENV strains are classified into four distinct serotypes (DENV1 to 4).^2^ Clinical manifestations range from asymptomatic or mild febrile illness to severe dengue hemorrhagic fever and dengue shock syndrome, posing substantial public health and economic burdens worldwide.^3–5^ The World Health Organization (WHO) 2025 fact sheet also reports spread into new areas, including parts of Europe and the Eastern Mediterranean, with autochthonous transmission.

Given these increasing global health threats, developing an effective vaccine against dengue is becoming more urgent. However, this endeavor is challenging mainly because of the risk of “antibody-dependent enhancement” (ADE).^6^ ADE occurs when non-neutralizing antibodies from a prior infection help the virus enter host cells, potentially exacerbating the disease. The currently recommended WHO vaccine has demonstrated suboptimal efficacy, particularly in DENV-naïve individuals, highlighting the urgent need for a safe and robust vaccine that provides balanced protection against all serotypes.

Virus-like particles (VLPs), which mimic viral antigenicity without containing the viral genome, represent a promising approach for vaccine development. They are advantageous due to their immunogenicity and potential for economical production across various systems. Several studies have shown the successful production of DENV VLPs in different expression systems, including plants,^7,8^ insect cells,^9,10^ bacteria,^11^ *Pichia pastoris*,^12–14^ silkworm larvae infected with recombinant baculoviruses,^15^ and mammalian cells.^16–18^ Although many DENV VLP vaccine candidates elicit neutralizing antibodies and/or protection in animal models, including nonhuman primates,^19^ electron microscopy-based structural analyses of DENV VLPs remain limited.

In materials terms, DENV VLPs can be viewed as protein–lipid nanoassemblies: a soft-matter shell in which a glycoprotein layer composed of precursor membrane (prM) and envelope (E) proteins is organized over a lipid bilayer. This framing emphasizes how composition (for example, lipids and N-glycans) and quaternary arrangement govern mesoscale mechanics, epitope display, and stability—key levers for rational nanoimmunogen design. Unlike rigid crystalline nanoparticles, VLP nanoassemblies are dynamic; their maturation involves large-scale, symmetry-constrained rearrangements that couple protein interfaces to membrane mechanics. A mechanistic understanding of these nanoassemblies may help inform design strategies aimed at enhancing the stability and minimizing conditions that favor ADE.

Our previous studies^20^ have demonstrated that mature VLPs derived from dengue serotype 2 (mD2VLPs) can generate high and broad neutralizing antibodies against all four serotypes in mouse immunization experiments. We demonstrated that these VLPs have the potential to be vaccine candidates for preventing DENV-induced diseases. Notably, the absence of the pr peptide in mD2VLPs reduced the ADE effect, thus increasing their suitability as safe vaccine candidates. Further, we have also successfully engineered chimeric mature DENV VLPs with enhanced secretion, stability, and immunogenicity using an integrative rational design approach.^21^ Together, these observations motivate a structure-focused examination of the immature VLP state and its relationship to maturation as a foundation for design.

DENV maturation is facilitated by furin, a protease primarily located in the trans-Golgi network (TGN). During this process, genomic RNA bound to capsid proteins forms a nucleocapsid core within the endoplasmic reticulum (ER), which acquires an envelope featuring 60 icosahedrally arranged trimeric spikes of (prM–E)_3_, leading to the formation of an immature virus measuring ∼60 nm in diameter.^22–24^ As the virus transits the acidic compartments of the secretory pathway, significant conformational changes enable furin to cleave prM into prM and M. This cleavage transforms the 60 prM–E trimers into a smooth lattice composed of 180 E–M heterodimers arranged on the surface as 90 E dimers. After furin cleavage, the pr remains bound to the E protein, effectively preventing premature membrane fusion until it is shed during the mature virus’s release through exocytosis. The smooth mature virion (∼50 nm) contains 180 copies each of E and M proteins; E forms 90 head-to-tail homodimers, and three E dimers align in parallel to form a raft structure.^22,25^

Despite the advances in cryo-EM, which have elucidated the static structural “end points” along the pathway from immature particles with 60 prominent spikes to the mature virion’s smooth surface composed of 90 dimers,^26^ the maturation pathway, intermediates, and dynamics for VLPs remain poorly characterized. A recent structural study of small dengue VLPs reported that maturation produced modest and irregular surface reorganization, suggesting that the conformational transition in VLPs is less extensive than in virions, although no mechanistic explanation for this reduced rearrangement was proposed.^27^ Here, building on our previous work on mD2VLP,^21^ we resolved the structure and organization of immature DENV-2 VLPs (imD2VLPs) produced in Chinese hamster ovary (CHO-K1) cells and analyzed their dynamics as protein–lipid nanoassemblies. The cryo-EM structure revealed a distinctive spiky surface consistent with 20 trimeric prM–E protomers on a *T* = 1 shell, contrasting with the 60-trimer immature virion. Using targeted molecular dynamics (TMD) to assess steric feasibility during the maturation process, we explored whether prM–E protomer rearrangement could accommodate the reorganization of trimeric prM–E spikes into E–M dimers under *T* = 1 constraints. The simulated transitions are guided between experimentally resolved end point structures. They are interpreted here as defining sterically and geometrically feasible rearrangements rather than uniquely determined maturation pathways. Both the virion and the VLP may undergo similar underlying protein motions; the resulting maturation-associated rearrangements in the VLP are substantially smaller in magnitude than those required for the virion due to differences in size, presence of nucleocapsid core, prM–E–lipid envelope interactions *etc*. MD simulations indicate strong interactions between the E proteins and anionic PS lipids, which contribute to stabilization of the immature particle within the limits of the model. At low pH, partial protonation of the PS phosphate group weakens these interactions, which may facilitate reorganization toward the mature state. Simulations qualitatively illustrate how lipid composition, lipid number, and N-glycan dynamics can influence overall particle morphology and associated cryo-EM density features. Overall, we provide a structure-based framework for imD2VLP nanoassembly, describe sterically feasible structural transitions associated with maturation, and outline process-relevant, testable hypotheses focused on lattice geometry and maturation control.

## Results and Discussion

### Establishment of a Stable Cell Clone Continuously Releasing prM-E Containing VLPs

In our previous study,^20^ we investigated the production of mD2VLPs via transient transfection of COS-1 cells with plasmids encoding M and E proteins. These particles induced the high titers of neutralizing antibodies against dengue virus following mD2VLP immunization in mice. Cryo-EM analysis of the secreted particles revealed that the mD2VLPs displayed *T* = 1 icosahedral symmetry and exposed highly overlapping cryptic neutralizing epitopes, supporting their potential as a vaccine candidate.^20^ Building on this, we sought to establish a mammalian cell clone that continuously releases imD2VLPs, providing a more efficient production system than transient transfection.

Inspired by the work of Konishi *et al.*, who engineered a CHO-K1 cell line to produce high yields of Japanese encephalitis viral (JEV) antigens by modifying the prM/M cleavage site to reduce fusion-mediated cytotoxicity,^28^ we adopted a similar strategy for DENV-2. CHO-K1 cells were transfected with a cDNA construct encoding DENV prM–E, in which the E sequence was chimeric (80% DENV-2 and 20% JEV), and the prM contained a mutated furin-cleavage site, to stably express prM–E proteins and favor immature particle formation. This design inhibited the furin-mediated processing of prM and thereby promoted the assembly of imD2VLPs. Transmission electron microscopy (TEM) performed on thin sections of Epon-embedded CHO-K1 cells at 72 h post-expression revealed spherical, electron-dense particles contiguous with the plasma membrane (Figure 1A). Antigen-capture ELISA showed sustained secretion of E antigen in the culture supernatant across four sequential harvests at 6-day intervals, indicating that the engineered CHO-K1 clones continuously released imD2VLPs (Figure 1B).

**Figure 1:**
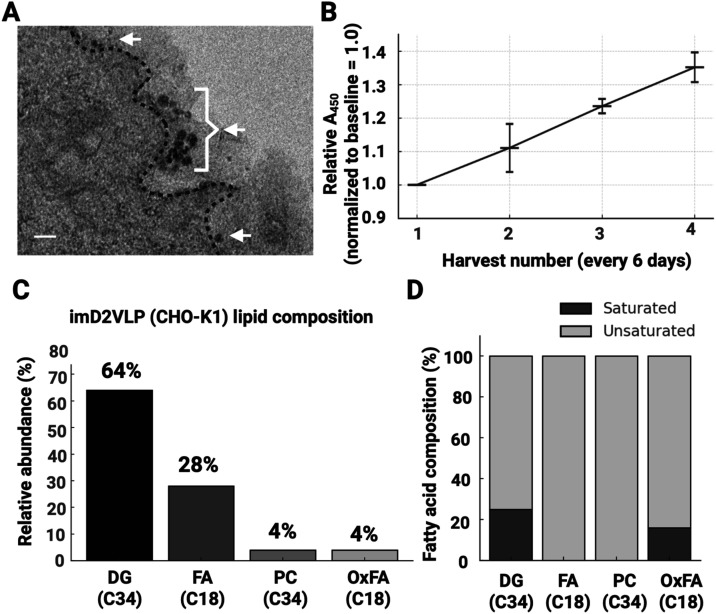
Production and release of immature DENV-2 virus-like particles (imD2VLPs) from CHO-K1 cells. (A) Transmission electron micrograph of a resin-embedded ultrathin section collected 72 h postexpression, showing electron-dense spherical particles (arrowheads) contiguous with the plasma membrane (PM; dashed line). The bracket marks a representative budding profile. Scale bar, 50 nm. (B) Relative ELISA signal (A_450_) across four sequential harvests at 6-day intervals from confluent cultures, normalized to harvest 1. Points show mean±SEM; *n* = 2 biological replicates per harvest; blank-subtracted. A linear trend indicates sustained release (slope = 0.116 per harvest; *R* ^2^ = 0.999). (C) Lipid composition of purified imD2VLPs measured by LC–MS: relative abundance of diacylglycerol (DG), free fatty acids (FA), phosphatidylcholine (PC), and oxidized fatty acids (OxFA). Dominant total acyl-chain carbon numbers are indicated below each class (DG, C34; FA, C18; PC, C34; OxFA, C18). (D) Fatty-acid saturation within each class (black, saturated; gray, unsaturated): DG 25/75%, FA 0/100%, PC 0/100%, OxFA 16/84%. These measurements informed the DG-dominant and PL-dominant vesicle compositions used in coarse-grained simulations (Figure 4).

We first quantified the lipid composition of purified imD2VLPs by liquid chromatography–mass spectrometry (LC–MS), as described previously.^21^ Diacylglycerol (DG) and free fatty acids (FA) were predominant lipid classes, with smaller amounts of phosphatidylcholine (PC) and oxidized fatty acids (OxFA); DG and PC were enriched in C34 species, whereas FA and OxFA were mainly C18 species (Figure 1C). Within each class, unsaturated acyl chains predominated (DG 75%, FA 100%, PC 100%, and OxFA 84%) (Figure 1D). These LC–MS measurements guided the composition of the coarse-grained (CG) vesicles (DG-dominant versus phospholipid (PL)-dominant) and the total lipid numbers used in the whole immature VLP CG models.

### Characterization of imD2VLPs

To further characterize the imD2VLPs, we purified them via sucrose gradient centrifugation and performed cryo-EM analysis. Cryo-EM imaging revealed that the concentrated imD2VLPs were monodispersed (Figure 2), displayed a rougher surface than mD2VLPs,^20^ and appeared smaller than immature flaviviral virions.^29,30^ Reference-free 2D classification of imD2VLPs resolved three populations based on morphology and size (Figure 2; Figure S1). The majority (∼76%) comprised particles with radially distributed peripheral densities surrounding a near-spherical lipid bilayer. A second population (∼22%) exhibited surface irregularities with nonspherical bilayers, whereas a minority (∼2%) comprised double-membrane vesicles of ∼40–52 nm in diameter.

**Figure 2:**
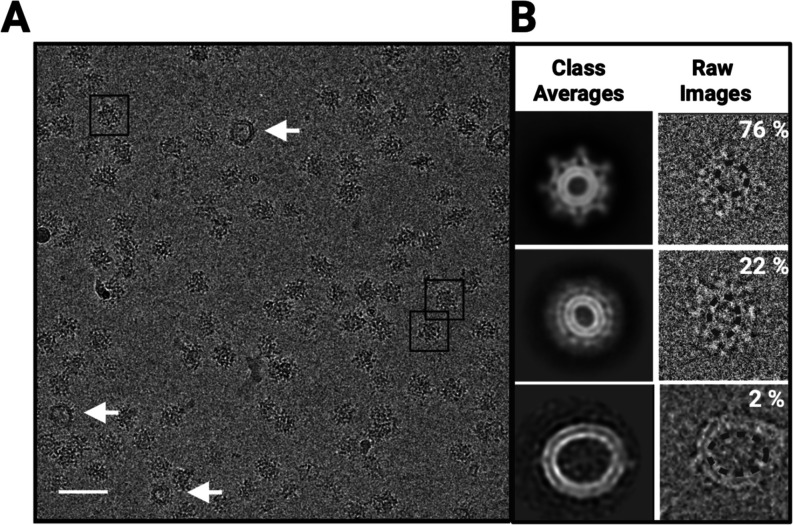
Cryo-EM micrographs and reference-free 2D classification of imD2VLPs. (A) A representative cryo-EM micrograph of concentrated particles on vitreous ice shows well-separated particles. Boxed regions mark representative particle picks used for downstream processing, and arrows indicate representative double-membrane vesicles. Scale bar, 50 nm. (B) Reference-free 2D class averages (left) and their corresponding raw particle images (right) resolve three image classes under no imposed symmetry. Based on assignments after 2D classification, these classes comprise ∼76, ∼22, and ∼2% of particles, respectively: the particles with radially distributed peripheral densities surrounding a near-spherical lipid bilayer (top); the particles with smeared or less well-defined peripheral densities and nonspherical bilayer contours (middle); the double-membrane vesicles (bottom). Dashed circles delineate the inner lipid bilayer.

The cryo-EM reconstruction of the predominant imD2VLP population reached an average resolution of 9.2 Å, revealing a multilayer architecture featuring 20 prominent spikes in a *T* = 1 arrangement (Figure 3). The spiky VLPs measured ∼35 nm in diameter, larger than the ∼31 nm observed in mD2VLPs.^20^ Rigid-body fitting of the trimeric prM–E from the immature DENV-2 virion (PDB: 4B03) into the 9.2 Å imD2VLP map indicated that each spike comprises three prM–E heterodimers capped by three pr domains. The spikes display local 3-fold symmetry, consistent with immature flavivirus structures, and the pr/M furin-cleavage site is positioned within the spike interior^26,29–31^ (Movie S1). In this fitted orientation, the cleaved pr peptide forms a cap over the E spike apex, while the fusion loop-proximal region of DII and much of DIII are oriented toward the spike interior beneath the pr cap, consistent with the reduced level of exposure of these regions. This arrangement agreed with our previous observation where imD2VLPs elicited lower levels of fusion-loop-specific and DIII conformation-independent antibodies than mD2VLPs.^20^ These observations defined the overall spike architecture of imD2VLPs and provided a structural basis for subsequent analyses of the lattice geometry and process-relevant features.

**Figure 3:**
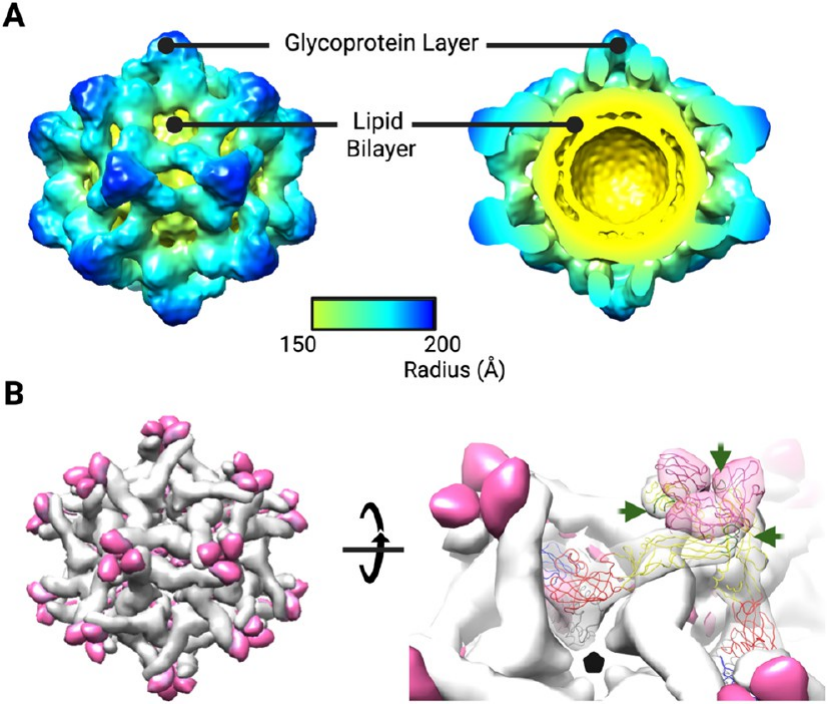
Cryo-EM structure of the immature DENV-2 virus-like particles (imD2VLPs). (A) The sharpened cryo-EM map of the imD2VLP contoured at 2.1σ, viewed along a 2-fold axis (left). The map is radially colored to indicate particle diameter. A cross-section (right) reveals a multilayered architecture comprising the glycoprotein shell (blue–green) and the lipid bilayer (yellow). The color bar denotes radius. (B) A surface rendering (gray) with fitted surface proteins (PDB: 4B03) is consistent with a *T* = 1 icosahedral arrangement. The surface spike is composed of a (prM/E)_3_ trimer, with E heterodimerized with prM. The cleaved pr peptide (magenta) caps the E protein (left panel). A closer view (right panel) shows the fitted proteins near a 5-fold axis (pentagon). Rigid-body fitting of the immature DENV-2 prM–E trimer into the map yields a map-model cross-correlation of 0.863. In this domain-level placement, the cleaved pr peptide caps the E-spike apex, with the fusion loop-proximal region of DII positioned beneath the pr cap and DIII oriented toward the spike interior. Because the fitting is performed at the domain level, this interpretation is qualitative; nevertheless, this placement is broadly consistent with prior observations in mD2VLPs showing weak targeting of the fusion loop by polyclonal sera and comparatively low responses to conformation-independent epitopes in DIII.^20^ E domains are colored (DI, red; DII, yellow; DIII, blue) and the fusion loop is indicated in green and pointed by green arrows.

### Coarse-Grained Simulations Reveal Intrinsic Dynamics of imD2VLPs and Lipid Composition-Specific Remodeling of Nanoassemblies

To explore the intrinsic dynamics of imD2VLPs, we built several CG models of entire imD2VLP particles consisting of 20 trimeric protomers of the prM-E protein. Due to the low resolution of the cryo-EM map, the stem-helix (SH) and transmembrane (TM) regions (SHTM) in the imD2VLP structure were not resolved and were modeled based on available experimental structural data, as described in Methods. These were embedded in lipid vesicles of ∼210 Å in diameter, positioned according to the cryo-EM density map (Figure 4A–C). We used two different experimentally determined lipid compositions with various numbers of lipids in different possible vesicle models to assess the stability of imD2VLPs and compared the results with cryo-EM micrographs (Figure 4B–C). We studied PL-dominant vesicles containing POPC:POPE:POPS in a 60:30:10 ratio^32,33^ (VLP_PL_) as well as DG-dominant vesicles composed of DG:SAPC:FA in a 56:28:6 ratio (VLP_DG_) based on previous lipidomics of COS-1-derived particles.^21^ We tested lipid vesicles with two distinct total numbers of lipids for both the PL and DG-dominant lipid compositions. This resulted in four systems: VLP_PL_-1 (∼2000 lipids in total), VLP_PL_-2 (∼1000 lipids in total), VLP_DG_-1 (∼2000 lipids in total), and VLP_DG_-2 (∼1000 lipids in total), as shown in Figure 4C. For each system, we performed 400 ns long equilibration simulation with the positions of protein backbone atoms restrained, which was followed by three independent 2000 ns unrestrained simulations.

**Figure 4:**
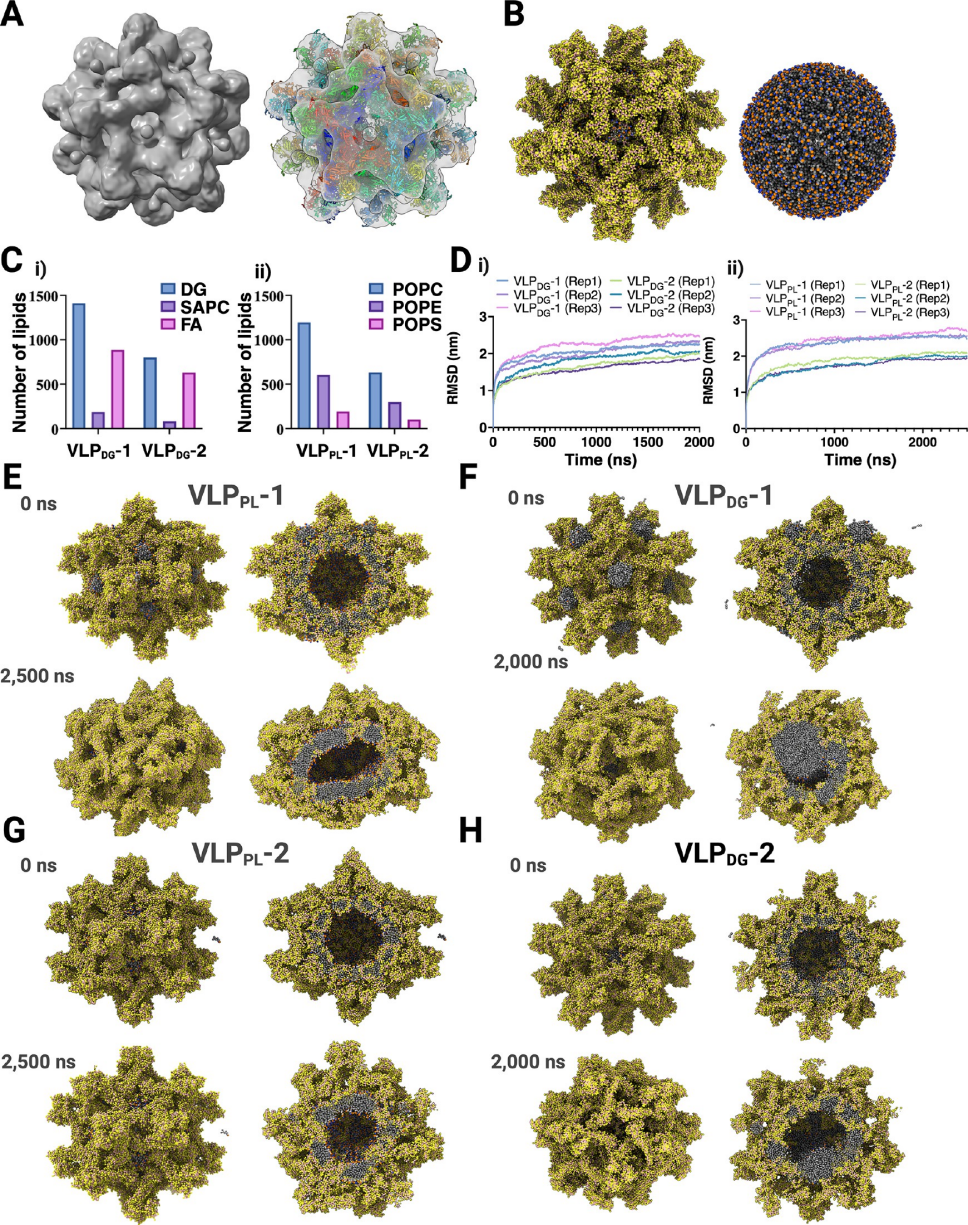
Coarse-grained MD simulations of imD2VLPs. (A) The cryo-EM map (surface representation) is shown together with atomistic coordinates of 20 trimeric units of prM-E protein (PDB: 4B03) that were rigid-body fitted into the density (right panel). The E–prM protein is shown in cartoon representation with distinctive colors for each monomer. (B) The initial coarse-grained (CG) model of fully assembled imD2VLPs (left panel) was shown together with the corresponding lipid vesicle (right panel). (C) Lipid composition and counts were plotted for the indicated constructs: (i) DG-dominant systems (VLP_DG_-1, VLP_DG_-2) showing DG, SAPC, and FA composition; (ii) PL-dominant systems (VLP_PL_-1, VLP_PL_-2) showing POPC, POPE, and POPS composition. (D) Backbone-bead RMSD of the VLP proteins relative to the starting experimental model is shown as a function of simulation time: (i) DG-dominant systems; (ii) PL-dominant systems. RMSDs are shown for all independent replicates (Rep1–Rep3). (E–H) Initial (0 ns) and final snapshots of the immature VLP simulations were shown for (E) VLP_PL_-1, (F) VLP_DG_-1, (G) VLP_PL_-2, and (H) VLP_DG_-2 at the indicated times (0 ns and 2000–2500 ns). In (B) and (E–H**)**, the protein and lipids are shown as spheres (protein, yellow; lipid tails, gray; lipid headgroups, red).

The root-mean-square deviation (RMSD) of all protein backbone beads for each VLP construct exhibited values ranging between ∼1.5–2.5 nm with respect to the experimental structure by the end of the simulation time (Figure 4D). Among all of the VLP simulation systems, VLP_PL_ constructs showed lower RMSD than VLP_DG_ systems. VLP constructs with a smaller number of lipid molecules (VLP_PL_-2 and VLP_DG_-2) tended to be more stable in comparison to systems with a higher number of lipids (VLP_PL_-1 and VLP_DG_-1), while VLP_PL_-2 was the most stable among all systems (Figure 4E–H). In the case of the VLP_DG_ simulation trajectories, the formation of a lipid bulge in the lipid envelope was observed among all DG-containing VLP systems. This is in agreement with previous studies in which the accumulation of DG lipid molecules in the space between the lipid bilayer (lipid lens) was observed, arising from the trans-bilayer activity of DG-rich lipid vesicles.^34,35^ Lipid lens formation was observed within the first 200 ns of the unrestrained production run (Figure 4H). The imD2VLP cryo-EM structure was obtained with icosahedral symmetry constraints and showed a spherical lipid bilayer (Figures 3 and 4). In this case, we compared the more stable VLP_PL_ constructs obtained from our simulations. The final frames of VLP_PL_-1 and VLP_PL_-2 exhibited ellipsoidal and spherical shapes, respectively, consistent with the lipid molecule number being one factor that may contribute to the conformational heterogeneity observed in cryo-EM micrographs (Figure 4E–G). Analysis of the simulated dynamics of the SH and TM regions showed considerable differences between those of VLP_PL_-1 and VLP_PL_-2. The distribution of solvent accessible surface area (SASA) for each E protein monomer showed variations among SH and TM regions, both within and between the respective VLP_PL_ MD simulations (Figure 5A). The number of contacts between the lipid envelope and each SH region of the E protein showed a significant difference between VLP_PL_-1 and VLP_PL_-2 due to the different morphology that VLP constructs adopt during simulations (Figure 5B). These results suggest that the SH and TM regions adopt heterogeneous, independent orientations, and therefore their densities are likely averaged out during cryo-EM reconstruction, resulting in weak or unresolved density for these regions. Further, we calculated and compared other biophysical variables from VLP_PL_-1 and VLP_PL_-2 trajectories such as root-mean-square fluctuations (RMSF) and prM or E protein domain SASA *etc* (Figure S2).

**Figure 5:**
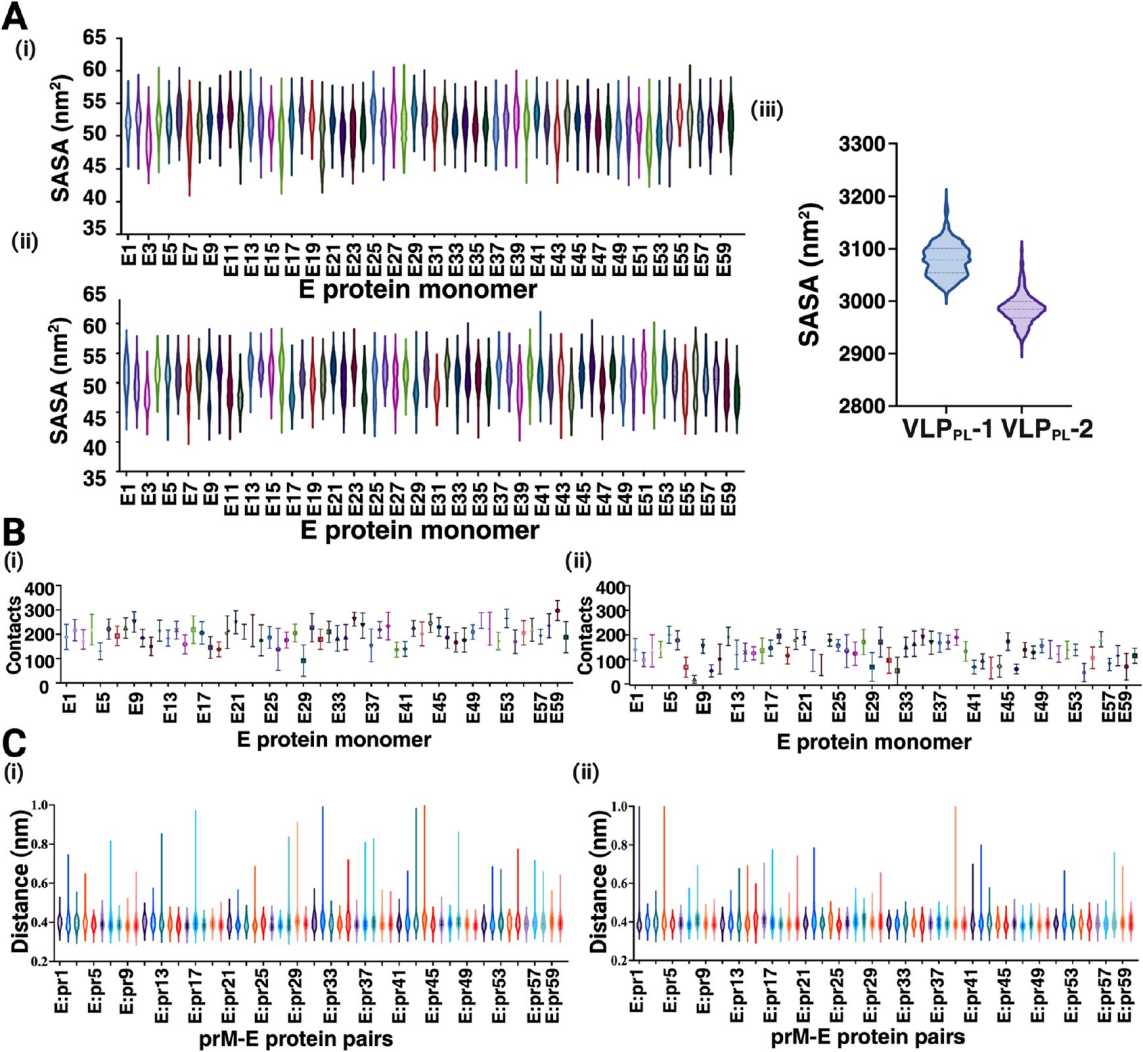
Dynamics of imD2VLPs in two PL-dominant lipid vesicles. (A) Solvent accessible surface area (SASA) of the stem-helix (SH) region (residues 396–450) was summarized as violin plots for each E-protein monomer in (i) VLP_PL_-1 and (ii) VLP_PL_-2; (iii) A combined violin plot of SH SASA for both VLP systems (VLP_PL_-1 and VLP_PL_-2). (B) SH–lipid contacts were averaged for (i) VLP_PL_-1 or (ii) VLP_PL_-2 using a 0.6 nm cutoff distance. Error bars correspond to standard deviations. (C) Violin plots of the minimum distance between E-DII and the interacting pr peptides for each of the 60 prM–E heterodimers in (i) VLP_PL_-1 or (ii) VLP_PL_-2. All distributions and summary statistics were computed across three independent simulation replicates.

Principal component analysis (PCA) was performed on the concatenated triplicate simulation trajectories of each VLP_PL_ system and revealed the dominant motions exhibited in the VLP_PL_-1 and VLP_PL_-2 trajectories. Comparison of the most dominant motions (PCA1 and PCA2), which accounted for >70% of the covariance, revealed a higher magnitude of structural changes in VLP_PL_-1 compared to VLP_PL_-2, accompanied by a loss of spherical shape (Figure S3A–D). We also calculated the minimum distance between each pr peptide and the E-protein DII per prM–E heterodimer, which increased more frequently in the VLP_PL_-1 system (Figure 5C), because more pr molecules dissociated due to the loss of sphericity (Figure S3E). Collectively, the simulations suggest a composition-dependent stability regime for imD2VLP nanoassemblies, in which approximately ∼1000 lipids and a PL-dominant membrane are associated with enhanced shape robustness and reduced pr dissociation during long-time scale dynamics. Further, we calculated the SHTM per-residue interactions with each lipid type in both immature and mature DENV VLPs using CG-MD simulations from this and our previous study of mature VLP (Figure S4A–B). In both systems, the lipid-interaction profiles showed strong SHTM–POPS contacts, consistent with observations from native virion simulations.^36^ These electrostatic interactions between the E-protein SHTM region and POPS contribute to the stabilization of the immature particle within the limits of the model. At low pH, partial protonation of the PS phosphate group weakens these interactions, which may facilitate reorganization toward the mature state. We also compared domain-specific SASA for the E protein in immature and mature CG-MD simulations using VLP_PL_-1 and VLP_PL_-2 CG models (Figure S4C). The spiky immature CG-MD trajectories clearly show a higher magnitude of DII and DIII solvent accessibility in both VLP CG models trajectories, indicating pronounced exposure of ADE-causing epitopes such as the fusion-loop epitope (FLE) in DII. Multiple studies^37–39^ have reported that immature virions induce ADE through antibodies such as FLE antibodies, and similarly, the enhanced solvent exposure of DII observed in immature VLP may induce ADE-causing antibodies akin to those elicited by native immature virion. To explore whether a structural rearrangement could sterically accommodate the differences observed between experimental structures, we employed targeted molecular dynamics (TMD) as a hypothesis-generating tool.

### Targeted Molecular Dynamics (TMD) Simulations Explored Sterically Feasible Rearrangements Associated with VLP Maturation

Similar to the DENV virion, the imD2VLPs are expected to undergo large-scale conformational changes during maturation. The immature VLP transitions from a spiky *T* = 1 state bearing 20 trimeric prM–E spikes to a smooth *T* = 1 state with 30 E–E dimers (with M proteins lying beneath the E ectodomains). This is triggered by lowered pH and furin protease cleavage inside the host cell.^40,41^ To explore sterically feasible rearrangements compatible with the VLP maturation process, we employed TMD simulations. In this approach, an additional bias is added to the total energy as a harmonic potential governed by the RMSD with respect to the target coordinates without representing physical kinetics. The target RMSD evolves linearly over the simulation time and triggers the transition from the starting coordinates to the target structure.^42^ Here, we employed TMD simulations of the VLP_PL_-1 in the immature trimeric state, biased toward the smooth mature state.

As described in the Experimental section, steering forces were applied to specific E and M proteins to trigger the conformational transition. We superimposed the immature and mature VLP^20^ coordinates along the 5-fold symmetric axes and assigned each chain based on the lowest RMSD value between the initial (immature) and final (mature) conformation (Figure 6A). The protein backbone RMSD value between the two states corresponded to ∼6 nm. First, we assessed the influence of the strength of the RMSD bias applied to immature VLP beads. We tested four different force constants of the harmonic bias potential: 100, 500, 1000, and 10,000 kcal mol^–1^ Å^–2^ (named TMD-1, TMD-2, TMD-3, and TMD-4, respectively) and ran four independent 100 ns long TMD simulations. In all TMD simulations, the conformational transition from a trimeric spike to a dimeric smooth state could be achieved without steric clashes between E protein monomers (Figure 6B), while retaining a symmetrical particle shape. Also, we observed subtle changes in the diameter of the lipid envelope during the transition among all of the TMD simulations. It gradually changed from ∼250 to ∼242 Å over the simulation time across all the TMD simulations, in agreement with experimental values (∼245 Å) (Figure 6C).

**Figure 6:**
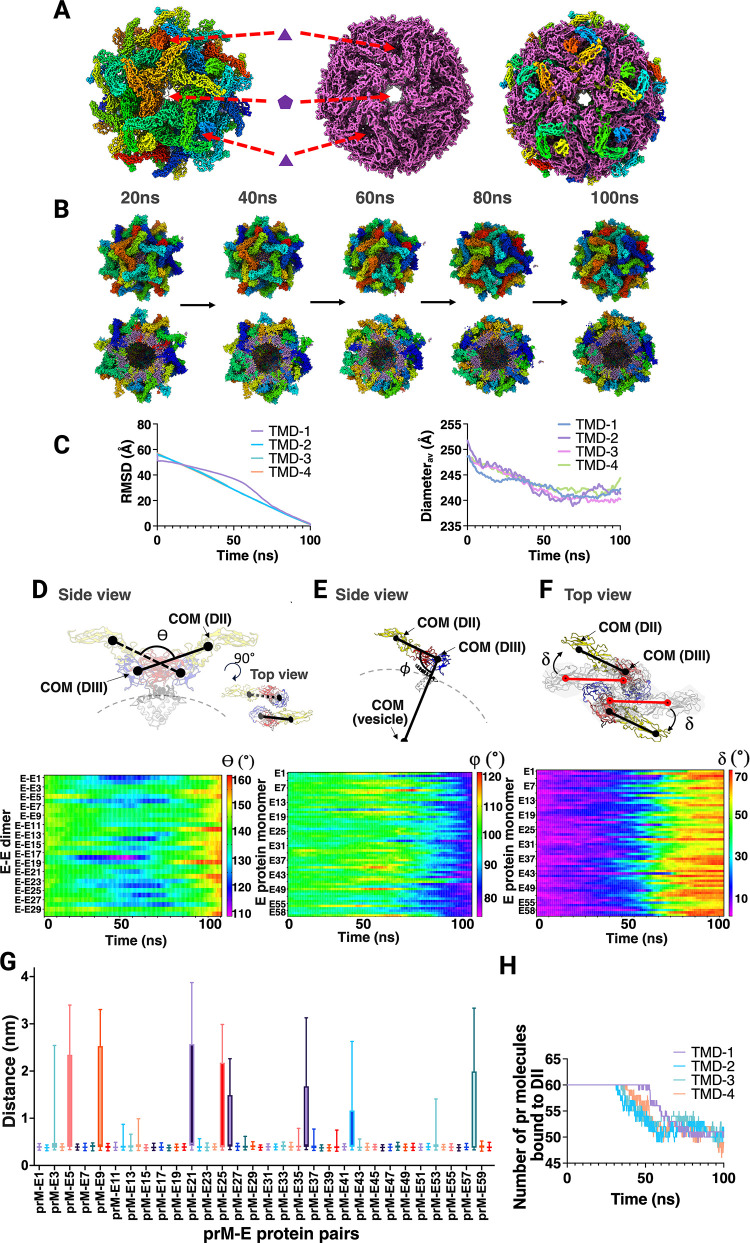
Targeted molecular dynamics simulations capture the immature-to-mature transition pathway of dengue VLPs. (A) Immature (left panel) and mature (middle panel: previous study)^20^ VLP structures were superimposed prior to any simulation. The corresponding five- and 3-fold symmetry axes are shown for both structures. Individual E-protein monomers from the immature and mature states were color-coded. On the right panel, E proteins from the mature (purple) and immature (rainbow) VLPs were superimposed along symmetry axes. (B) TMD-1 simulation snapshots showing the transition from spiky trimeric state to smooth dimeric conformation. The entire VLP (top panel) and its cross-section (bottom panel) of immature VLP at *t* = 20, 40, 60, 80, and 100 ns during the TMD simulation with distinct colors for each E protein and purple lipid beads. (C) RMSD of protein backbone beads with respect to the final mature VLP structure over the simulation time for different force constants (100, 500, 1000, and 10,000 kcal mol^–1^ Å^–2^) corresponds to TMD-1, TMD-2, TMD-3, and TMD-4, respectively (left panel). The VLP vesicle diameter over each TMD is shown on the right. (D) Cartoon representation of single unit of E protein dimer and angle ⊖, along with the heatmap showing ⊖ for all 30 pairs of E-dimers over the course of the TMD-1 simulation time (bottom panel). (E) Cartoon representation of E protein monomer and angle Φ, along with the heatmap showing changes in angle Φ for all 60 E proteins during the TMD-1 simulation time (bottom panel). (F) E protein dimers at *t* = 0 ns (gray) and their subsequent movements over the simulation time (colored) are shown in cartoon representation from the immature VLP. The vector connecting DII and III at *t* = 0 ns and at a later time point are shown in red and black, respectively, along with the angle δ. The heatmap (bottom panel) depicts changes in angle δ for all 60 E proteins during the TMD-1 simulation. In panels (D–F) proteins are shown as cartoons and domain-colored (DI, red; DII, yellow; DIII, blue), with the stem in black and the transmembrane (TM) region in white. (G) Box-and-whisker plots report the minimum distance between E-DII and the corresponding pr peptides for each prM–E heterodimer, averaged over the TMD-1 simulation. (H) The number of pr molecules within 0.6 nm of DII for all 60 prM-E protein monomers is shown over simulation time.

Overall, TMD simulations demonstrated the steric feasibility of a transition from a trimeric to a dimeric state via a “sliding-rotating” motion without any steric clashes among prM-E proteins (Movie S2). This is consistent with a recently proposed maturation model for DENV described by Duquerroy et al. using static endpoint icosahedral structures at low pH and neutral pH.^43^ To quantify this sliding and rotating motion, we calculated three distinctive angles of E protein monomers or dimers over the course of the simulation time (Figure 6, and the Methods section). All angles in the first frame corresponded to the experimentally determined cryo-EM structure, and subsequent changes reflected structural changes occurring during the TMD simulations (Table S3). Initial value (immature state) of angle ⊖ was averaged over the 30 pairs of E-dimers in the first frame and corresponded to 142±0.2°. After the transition, its value corresponded to a range between 148±0.3° and 160±0.1° in the final frame of the simulation (mature state) depending on the force constant used (Table S3). Interestingly, for almost all the E protein pairs and from all four TMD simulations, the ⊖ value gradually dropped to ∼120° from the initial value and started to increase in the latter part of the simulation time along with an increase in the number of E–E dimer interactions (Figure 6D; Figure S5A–B). This showed that during the sliding-rotating motion, the E-dimers performed a “scissor”-like motion during the transition from spiky immature to a spherical mature state. In the case of Φ, the angle gradually decreased from 98±0.2° to 82±2° in TMD-1 or to 74±0.6° in the case of TMD-4 (Table S3; Figure 6E; Figure S5C). It was noteworthy that the averaged ⊖ and Φ values for the whole imD2VLP from the final frame of TMD simulations showed values comparable to those of the mature VLP. In particular, the final state of TMD-4 simulations attained an identical ⊖ (∼160°) value averaged over all of the 30 dimeric units of mature VLP structure (Table S3). Finally, angle δ over the simulation time showed that each E protein monomer gradually adopted an angle of 65±5° to 68±4° with respect to the initial E protein orientation in the immature state (Table S3; Figure 6F; Figure S5D). We further calculated the minimum distance between the pr domain of prM and the DII of E across all prM–E pairs. This analysis showed that most prM–DII pairs remained in contact throughout the transition, with only occasional displacement events (Figure 6G–H; Figure S5E). The number of pr molecules that dissociated from E proteins was consistently ∼10 across all simulations, regardless of force constant, and was consistent with the unbiased MD simulations (Figure 5C; Figure S3E). Thus, the “pulling” rate (bias strength) did not affect pr-E engagement, indicating that the observed rearrangement is governed primarily by geometric compatibility within the nanoassembly. To assess the mechanical stability of the steered architectures, we performed a 500 ns of unrestrained simulations initiated from the final frames of independent TMD runs. During relaxation, the protein shell remained close to the target mature configuration with backbone RMSDs plateauing at ∼10–14 Å. In parallel, the lipid envelope exhibited stable mean radius and sphericity, indicating that lipid rearrangements induced during steering do not propagate into progressive membrane destabilization after removal of biasing forces (Figure S6). In the cellular context, previous studies on DENV have shown that exposure to low pH (∼6) in the TGN drives the structural changes that drive the prM-E protomers from spiky trimers into flat E dimers, which then tile the entire lipid envelope encasing the nucleocapsid. In the case of VLPs, our TMD simulations showed that this translation, *i.e.*, the sliding of each prM-E protomer accompanied by rotation, enables the formation of new quaternary contacts that assemble into the smaller *T* = 1 icosahedral particle.

### Empty Cryo-EM Density Maps Consistent with Dynamic Lipids and N-Glycans

During the equilibration simulations of both VLP_PL_ and VLP_DG_ systems, we observed that some lipid molecules spontaneously underwent transient protrusion events from the spherical lipid vesicle (Figure 7A) at the 5-fold symmetry axes in all simulations, irrespective of lipid composition and number of lipids used in the vesicle. Further, these lipid protrusion events were reversible, with reintegration into the lipid vesicle occurring within ∼10 ns of unrestrained production simulations. Nonetheless, 1–3 lipid molecules underwent outward excursions beyond the mean envelope boundary during the unrestrained simulations across all replicates. Upon closer inspection of the cryo-EM density map, we consistently observed a low-resolution density emerging out of the lipid vesicle density at all 5-fold axes (Figure 7Bi). For all structural interpretations, we used the consensus map reconstructed with imposed icosahedral symmetry, and the corresponding global gold-standard half-maps showed that this 5-fold-adjacent density is reproducible between the two independently refined half-sets (Figure 7Bii). Importantly, the membrane-proximal density feature is reproducible between independently refined half-maps (and in focused C1 half-map reconstructions), supporting the notion that it is not a reconstruction artifact but reflects a variably occupied, membrane-associated feature. To further assess the robustness of this feature, we performed focused C1 refinements with a soft mask centered on the 5-fold axis and reconstructed independent half-maps. In these focused maps, the membrane-proximal density was again observed in both half-maps at comparable contour levels (Figure S7). Consistently, local-resolution estimates calculated from the focused C1 half-maps within the 5-fold mask showed lower apparent resolution at the masked site than in the neighboring protein shell (Figure 7Biii), in agreement with reduced order and/or heterogeneous occupancy at this location. Consistent with this interpretation, a time-averaged three-dimensional lipid density from equilibrium MD simulations, colored by radius relative to the vesicle center of mass, reveals preferential outward deformation localized near the 5-fold vertices (Figure 7Biv), matching the position of the reproducible membrane-proximal density observed in the cryo-EM half-maps. Consistent with this spatial localization, 2D angular maps of the vesicle surface, expressed in spherical coordinates and averaged over triplicate unrestrained simulation trajectories, reveal lipid-type-dependent, spatially structured membrane deformations and lipid enrichment patterns rather than uniform radial fluctuations (Figure S8). Taken together, MD simulations support the observations from cryo-EM and suggest that this variably occupied, membrane-associated feature is compatible with an ensemble-averaged population of transient lipid excursions rather than a static structural element. To quantify the temporal behavior of lipid protrusion events, we monitored lipid headgroup excursions beyond the mean vesicle surface over time during both unbiased equilibration/production simulations and during TMD-driven maturation, followed by extended unrestrained relaxation. Across all conditions, lipid protrusion events were rare, transient, and reversible, involving only a small fraction of the total lipid population at any given time (typically <1–2% when normalized by lipid abundance; Figure S9). Importantly, comparable magnitudes and frequencies of protrusion were observed during unbiased simulations, TMD trajectories, and post-TMD unrestrained runs, indicating that these events are not induced by steering forces but instead reflect an intrinsic dynamic property of the lipid envelope. Lipids that transiently protruded from the vesicle surface frequently reintegrated into the membrane on nanosecond time scales, and no progressive accumulation or envelope destabilization was observed.

**Figure 7:**
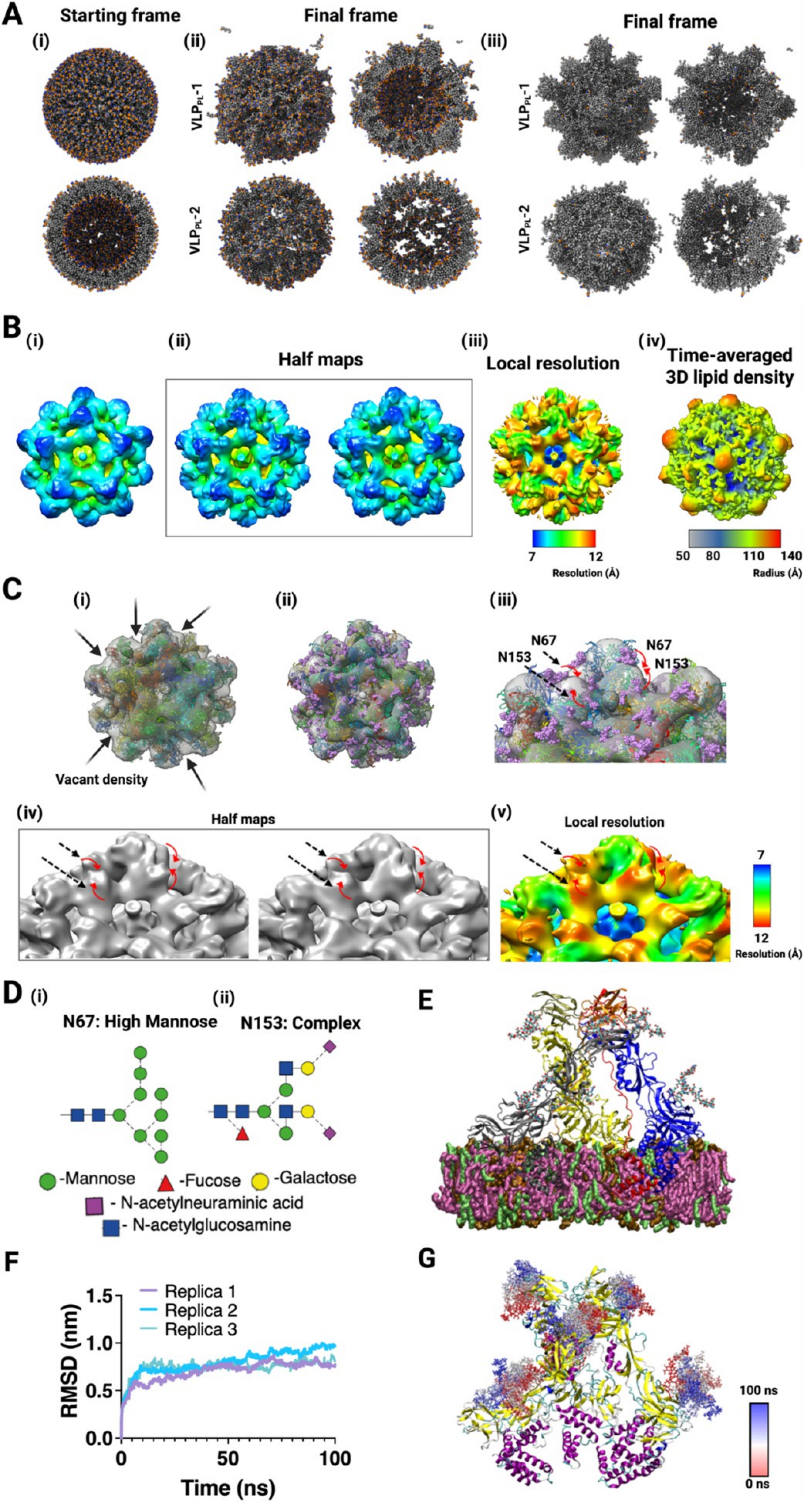
Cryo-EM and MD analyses reveal heterogeneous, membrane-proximal features and glycan-associated densities in imD2VLPs. (A) (i) A representative coarse-grained (CG) phospholipid-dominant (PL-dominant) vesicle model is shown together with its cross-section. (ii-iii) Snapshots of the VLP from the first and last frame of a 400 ns-long equilibrium simulation for VLP_PL_-1 (ii) and VLP_PL_-2 (iii) are shown for the entire particle and its cross-section. (B) Five-fold axis views throughout. (i) An icosahedrally averaged cryo-EM map of imD2VLP shown in surface representation, contoured at 0.27 σ. (ii) Half-map reconstructions using a soft mask centered on the 5-fold axis, showing reproducible membrane-proximal density at the inner leaflet near the axis (arrows) in both half-maps, consistent with a variably occupied membrane-associated feature. (iii) Local resolution estimated from the half-maps using the same mask and analysis settings. The masked region at the 5-fold site shows lower apparent resolution than the surrounding shell, consistent with reduced order and/or heterogeneous occupancy at this location; we therefore describe this signal as a variably present, membrane-associated density. (iv) Time-averaged 3D lipid density from equilibrium MD simulation trajectory of lipid envelope of VLP_PL_-1. The density map is colored by radius from the vesicle center of mass. Red regions are preferentially localized near the 5-fold vertices, consistent with the reproducible membrane-proximal density observed in the cryo-EM half-maps. (C) (i) A glycan-free and (ii) a glycosylated atomistic model of imD2VLP was fitted into the cryo-EM density map with unoccupied cryo-EM density regions highlighted. (iii) A close-up of two glycosylated trimeric spikes is shown. (iv) Half-map reconstructions contoured at the same global threshold as the consensus map, showing that the site-adjacent envelopes near N67 and N153 reappear in both independently refined half-maps (arrows), supporting reproducibility of these features. At 9.2 Å, we therefore describe them conservatively as glycan-compatible densities. (v) Local-resolution map calculated from the half-maps using masks centered on N67 and N153, revealing lower apparent resolution at these site-adjacent envelopes than in the neighboring E-protein density, consistent with conformational variability and/or partial occupancy at these positions. (D) Glycan composition and linkages were depicted at N67 (high-mannose) and N153 (complex-type). (E) Initial coordinates of a trimeric, glycosylated prM–E assembly embedded in a diacylglycerol-dominant (DG-dominant) lipid bilayer are shown. Proteins are shown as cartoons (E, gray; prM, magenta), and lipids are shown as a surface representation (lipid bilayer, orange). (F) Backbone beads RMSD of the proteins was plotted over three independent simulation replicas. (G) The starting structure (*t* = 0 ns) of the trimeric prM–E assembly was shown together with snapshots of the N67 and N153 glycans sampled every 10 ns over 100 ns (sticks colored from blue at *t* = 0 to red at *t* = 100 ns). Each protein molecule is shown as a cartoon and colored by secondary structure (β-sheets, yellow; α-helices, magenta; loops or unstructured regions, cyan).

A second region of unoccupied cryo-EM density appeared after fitting the atomistic VLP model to the experimental map (Figure 7Ci–iii). For global interpretation, we used the icosahedral averaged consensus map. The corresponding gold-standard half-maps showed that the site-adjacent envelope is reproducible between the two independently refined halves (Figure 7Civ). To further assess the robustness of this feature, we carried out focused C1 refinements with a soft mask encompassing the N67/N153 neighborhoods and reconstructed half-maps from the same particle half-sets. In these focused reconstructions, site-adjacent density was again observed in both half-maps at a single contour level (Figure S7).

Local-resolution estimates calculated from the focused C1 half-maps within masks centered on N67 and N153 showed lower apparent resolution in the site-adjacent envelope than in the neighboring protein density (Figure 7Cv**)**, consistent with a more mobile and/or partially occupied moiety at these sites. This region of the map was juxtaposed to the N67 and N153 sites of E-protein trimers (Figures 7Di–ii). We modeled experimentally informed glycans^44^ on each protein trimer and fitted these coordinates into all-atom VLP coordinates (Figure 7E). The modeled glycans overlapped with and substantially filled the previously unassigned density in the cryo-EM map. The all-atom MD simulations of glycosylated trimeric prM–E protein embedded in the lipid bilayer containing DG:PC:FA (56:28:6) revealed highly dynamic glycans at both N67 and N153 sites with a large average volume occupied (Figure 7F–G; Movie S3). In summary, the MD observations together with the cryo-EM map analyses support our interpretation of the previously unassigned densities in the reconstruction as arising from transient, heterogeneously occupied lipid excursions near the 5-fold axes as well as conformationally dynamic N-glycans at N67 and N153.

### Discussion of Structural and Simulation Findings

In our previous work, we elucidated, characterized, and engineered mD2VLPs as nanoassemblies, showing that their ordered architectures exposed cryptic neutralizing epitopes and elicited broad protection in mice.^20,21^ Building on this platform, we now resolved the structure of imD2VLPs and characterized sterically feasible large-scale conformational rearrangements associated with maturation as well as the intrinsic nanomechanics that govern particle stability.

A central question was how low the pH in the TGN induces the necessary conformational rearrangements in the prM–E spike. Although the ∼9 Å resolution cryo-EM map limited atomic-level interpretation, integrating cryo-EM data with CG simulations provided critical insights into the dynamic nature of VLP maturation. The simulations demonstrated the steric feasibility of a sliding-rotating motion of E protein protomers during the transition from an immature trimeric state to a mature dimeric state (Figure 8; Movie S2), offering a plausible structural bridge between the two nanoarchitectures. Because immature particles have been associated with ADE *in vivo*, a structural description of sterically feasible rearrangements may help generate hypotheses for future immunogen design while explicitly acknowledging that immunogenicity was not evaluated in this study.

**Figure 8:**
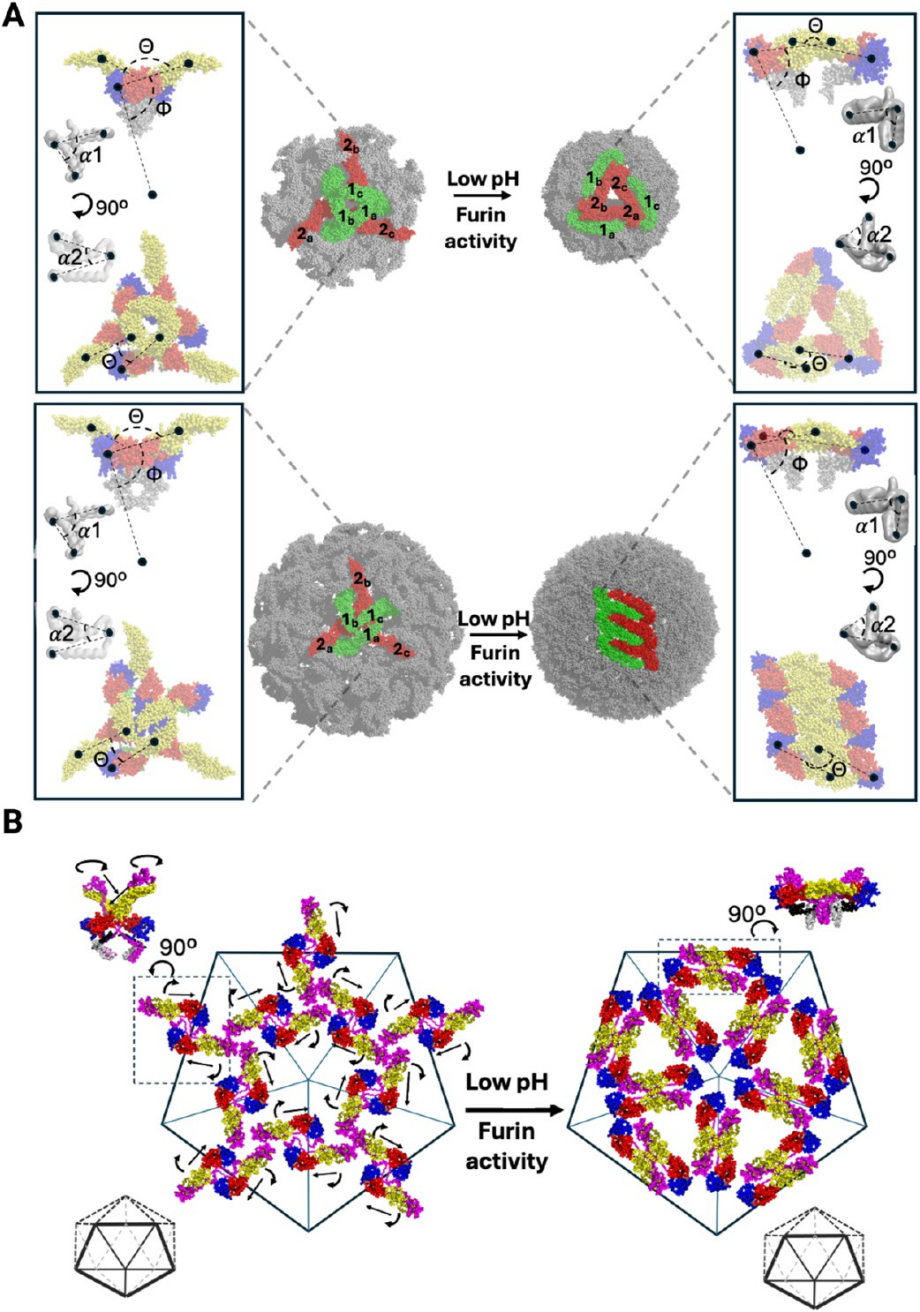
Conceptual framework relating immature and mature dengue VLP and virion organizations, based on end point-constrained TMD and experimental end point structures. (A) Schematic illustration of the quaternary organization of immature and mature dengue VLP (top panel) and virion (bottom panel), composed of 60 (*T* = 1) and 180 (*T* = 3) E-protein monomers, respectively. Dimeric units that undergo sliding-rotating motions during the transition from the spiky immature to the smooth mature conformation are highlighted in green and red and labeled as 1 or 2, with E-protein pairs carrying matching subscripts (a, b, or c). In the VLP, each immature trimer reorganizes through sterically feasible sliding-rotating motions to form a triangular arrangement composed of three mature dimers that assemble into the *T* = 1 lattice. In the virion, the immature trimeric units undergo a more complex structural transition to form the characteristic E-protein raft, comprising three parallel dimers, a process that also includes sliding-rotating motions. Insets show inter- and intra-E-protein angles for both immature and mature states (Table S4). (B) Schematic illustration of VLP prM–E units maturation at the 5-fold symmetry axes. Each particle contains 60 prM–E units arranged in *T* = 1 icosahedral symmetry, with 20 three-fold vertices and 12 five-fold vertices. The highlighted prM–E units at the 5-fold axis undergo coordinated sliding-rotating movements, as observed in the TMD simulations, transitioning from a spiky immature arrangement to a smooth, mature organization. Insets show side views of a dimeric prM–E unit in the immature and mature particles. In (A) and (B), the prM–E proteins are color-coded by domain (DI, red; DII, yellow; DIII, blue; stem, black; TM, white; prM, magenta) and shown in cartoon representation. This schematic illustrates geometric relationships and angular changes as one sterically feasible, geometry-compatible transition model, but does not represent validated mechanistic steps or a unique physical pathway.

Several structural studies have proposed diverse maturation mechanisms for flaviviruses. For the dengue virus, a recent cryo-EM analysis of VLP maturation reported a transition involving substantial structural rearrangements.^45^ Another dengue cryo-EM study proposed that the prM linker acts as a “drawstring” that rotates the ectodomains.^25^ The immature Spondweni virus structure suggests that histidine protonation in prM permits the E protein to transition from a strained trimeric spike to a smooth conformation.^46^ In contrast, the Binjari virus cryo-EM structure resolved the full prM linker at 4.4 Å and proposed a “pillar-collapse” mechanism.^29^ More recently, immature Zika virus was suggested to mature through a “latch-and-lock” mechanism driven by electrostatic and hydrophobic interactions between M and E proteins.^47^ Our observed sliding-rotating motion adds a geometry-driven, steric-compatibility perspective that explains how immature assemblies could transition in a *T* = 1 context without subunit clashes. These varied models highlight the fact that flavivirus maturation is multifaceted. Our findings add an additional dimension by suggesting that steric compatibility can facilitate a feasible maturation pathway for nanoassemblies.

The basic protomer of both the immature dengue virion and immature dengue VLPs is identical, a spiky trimeric prM–E. In the mature state, the virion exhibits a *T* = 3 surface comprising 90 E dimers arranged into 30 rafts, each forming a linear array of three head-to-tail E–E dimers resting above M proteins. In contrast, mature dengue VLPs present only 30 E dimers on a *T* = 1 shell. It is therefore plausible that immature virion spikes may undergo analogous sliding-rotating rearrangements, although the higher copy number of E dimers in virions likely introduces additional layers of cooperative interactions and lattice-specific constraints.^29,46,48^

During maturation, both dengue VLPs and full dengue virions undergo prM cleavage and low-pH-induced rearrangement of the prM–E trimeric spikes, but the resulting structural transitions differ substantially because the two particles assemble into distinct icosahedral organizations. The dengue virion adopts a *T* = 3 lattice in which each immature spike sits at an icosahedral 3-fold axis and, upon maturation, repacks into rafts of three parallel E dimers that generate the smooth mature surface. In contrast, dengue VLPs assemble into a *T* = 1 lattice and therefore lack the long-range symmetry and positional constraints required for extended raft formation. Instead, when the VLP spikes collapse, the neighboring monomers reorganize into local triangular clusters of three dimers rather than continuous planar rafts (Figure 8). As a result, the structural changes in *T* = 1 VLPs are less drastic and locally confined in comparison to *T* = 3 virions, as revealed by the analysis of absolute angular deviations between immature and mature states (Table S4). In agreement with previous studies,^36^ we also observe that anionic PS lipids interact most strongly with the E protein SHTM, in contrast to zwitterionic PC and PE. These stronger PS-SHTM interactions contribute to stabilization of the immature particle by anchoring the E proteins more effectively to the membrane. At low pH, the phosphate groups of PS become partially protonated, which weakens these interactions and may facilitate reorganization toward the mature E protein arrangement.

Although this work does not directly evaluate immune responses, prior comparisons of flaviviral VLPs and virions provide useful context for interpreting the lattice geometries observed here. Our dengue VLP cryo-EM analysis revealed a complex *T* = 1 assembly featuring 20 prominent spikes composed of trimeric prM–E heterodimers capped by globular pr domains. This architecture recapitulates key structural elements of immature flavivirus virions (Figure S10; Table S4). Geometry-coupled constraints in *T* = 1 lattices influence how quaternary epitopes are displayed. In our earlier work, mature DENV-2 VLPs exhibited greater solvent exposure around the E-dimer groove than virions, consistent with quaternary epitopes being more accessible on VLPs.^20^ Immunization studies similarly showed reduced prM-focused responses, broader neutralization, and *in vivo* protection.^20^ Human monoclonal antibody data further support geometry-dependent antigenic behavior; for example, K8b binds and neutralizes differently on *T* = 1 VLPs versus *T* = 3 virions.^49^ Together, these findings underscore that the VLP geometry can contribute to maturity-dependent antigenic remodeling.

We therefore frame our observations as envelope- and geometry-specific structural dynamics and maturation-relevant mechanistic constraints that may support rational VLP design rather than as validated rules for vaccine performance. Independent studies indicate that E-stem and membrane interactions influence VLP stability, secretion, and antigen recognition^21^ suggesting actionable parameters for future engineering. Accordingly, we propose several geometry-focused considerations: (i) maintaining stable E-dimer presentation on *T* = 1 lattices may preserve the E-dimer groove associated with quaternary neutralizing epitopes; (ii) modulating maturation parameters (pH, temperature, furin exposure) may reduce pr display and shift antibody responses, consistent with mD2VLP versus imD2VLP serology; (iii) fine-tuning E–lipid interactions to stabilize the mature VLP state within the relevant lipid composition; and (iv) matching antibody spacing and valency to the *T* = 1 lattice may optimize engagement patterns, as suggested by K8b.^49^ These considerations may help guide future engineering of enveloped VLPs.

Multiscale simulations enabled us to probe the inherent dynamics of imD2VLPs, providing qualitative insights into steric feasibility and envelope behavior. Our results suggest that imD2VLP stability depends on lipid composition: vesicles enriched in phospholipids and with fewer total lipids maintained a more spherical and robust morphology, whereas DG-dominant envelopes often generated lipid bulges that may affect membrane integrity. These findings indicate that lipid formulation is an important design parameter for scalable VLP manufacturing, analogous to optimizing a soft-matter scaffold for antigen display. Importantly, extended unrestrained simulations initiated from steered maturation end points demonstrate that both the protein shell and lipid envelope relax into stable dynamic regimes, with no evidence of progressive deformation following removal of biasing forces.

Another insight emerging from our work concerns the role of glycan flexibility. The diffuse densities observed near symmetry axes may reflect heterogeneous lipid protrusions or flexible glycan moieties rather than missing protein components or reconstruction artifacts. Time-resolved analyses further show that lipid protrusion events are rare, transient, and reversible, involving only a small fraction of the lipid population at any given time and occurring with comparable frequency under unbiased, TMD-driven, and post-TMD unrestrained conditions. These observations support an interpretation in which the membrane exhibits controlled, geometry-linked plasticity rather than persistent disorder or instability. Lipid protrusions at 5-fold axes during MD simulations, and their correspondence with cryo-EM density, highlight envelope-level, transient remodeling behavior that may be relevant to broad-spectrum antiviral design, including potential modulation of epitope accessibility and antibody engagement.

Importantly, the multiscale simulation framework applied here follows an approach previously validated on large enveloped viral assemblies,^21^ where experimentally constrained CG models were shown to remain stable over extended simulations and to yield mechanistically interpretable insights consistent with cryo-EM observations. This study has several limitations that should guide interpretation and future work. First, TMD simulations explore sterically feasible transitions constrained by experimental endpoints but do not capture activation energies or identify rate-limiting steps along the maturation pathway. Second, the 9.2 Å resolution of the immature VLP structure precludes an atomic-level interpretation of side-chain interactions. Third, while multiple lipid compositions were examined, the cellular membrane environment may include additional species and asymmetric distributions not captured here. Fourth, our VLP simulations do not include the nucleocapsid core (as VLPs inherently lack the nucleocapsid) present in virions, which may impose additional geometric constraints on maturation; therefore, limiting direct extrapolation from VLPs to virions. Future work incorporating cryo-electron tomography, subtomogram averaging, higher-resolution single-particle reconstruction, systematic variation of membrane composition, and *in vivo* immunogenicity testing will be important to further refine and validate these models.

## Conclusions

In summary, we established a mammalian production route for imD2VLPs and defined how their protein–lipid nanocages reorganize during maturation. The structure-dynamics framework reveals design-relevant relationships among lipid composition, lipid number, and quaternary-state stability, providing a foundation for hypothesis-driven engineering of dengue VLPs, including strategies aimed at limiting immature ADE-associated display states. Together, these findings establish a structure-dynamics framework for understanding flavivirus VLP maturation and provide experimentally constrained design parameters for future VLP engineering.

## Experimental Section

### Plasmid and Cells

The plasmid design was described previously.^20,50^ Briefly, we used the pVD2i backbone encoding prM and a chimeric E protein in which the DENV-2 ectodomain (E-DI to DIII; residues ∼1 and 395; Asian 1 genotype, strain 16681) was fused at the C terminus to the stem/transmembrane segment from Japanese encephalitis virus SA14–14–2 (residues ∼396–495). The designation “∼80% DENV-2/∼20% JEV” denotes this fixed domain boundary. This configuration preserves the DENV-2 ectodomain surface, while the JEV stem and transmembrane segment facilitate secretion and particle homogeneity in mammalian cells. To generate the uncleaved-prM construct, the minimal furin motif at the pr/M junction in pVD2i was mutated from REKR to REST by site-directed mutagenesis. The plasmid was transfected into CHO-K1-2-6-7 cells using lipopolyamine (Transfectam; Biosepra, Villeneuve-la-Garenne, France) according to the manufacturer’s instructions. Cells were cultivated in suspension in DMEM supplemented with 10% fetal bovine serum (FBS), 1% antibiotic–antimycotic, 1% MEM nonessential amino acids (NEAA), and 1% sodium pyruvate in a humidified atmosphere of 5% (v/v) CO_2_ at 37 °C. Cultures were maintained by routine seeding at low density.

### Harvesting and Purification of imD2VLPs

For production of imD2VLPs, 5.0 × 10^6^ CHO-K1-2-6-7 cells were seeded in a T150 flask and cultured overnight. After adherence, the medium was changed to serum-free maintenance medium (pH 7.4) containing 2% GlutaMAX-I, 1% sodium pyruvate, 1% MEM NEAA, 1% antibiotic–antimycotic, and 1% cholesterol, and the flask was transferred to 28 °C for 6 days. The culture supernatant was clarified by centrifugation at 10,000 rpm for 30 min at 4 °C. The clarified supernatant was layered onto a 20% (w/w) sucrose cushion and centrifuged at 25,000 rpm for 16 h at 4 °C. Pelleted VLPs were resuspended in TNE buffer (50 mM Tris-HCl, 100 mM NaCl, 1 mM EDTA) and recovered at 4 °C overnight. The recovered solution was applied to a 5–25% (w/w) sucrose gradient (prepared in TNE) and centrifuged at 25,000 rpm for 3 h at 4 °C in a Beckman SW-41 rotor. Fractions were collected, and E-antigen levels were determined by ELISA (see below). Fractions containing imD2VLPs were pooled, concentrated, and buffer-exchanged into TNE using Amicon Ultra 0.5 mL centrifugal filters (100 kDa MWCO; Merck Millipore, Germany).

### Antigen-Capture ELISA

A conventional antigen-capture enzyme-linked immunosorbent assay (ELISA) for quantifying imD2VLPs in culture supernatants was performed essentially as described.^20^ Briefly, rabbit anti-DENV-2 VLP serum in carbonate buffer (28.3 mM Na_2_CO_3_/71.4 mM NaHCO_3_) was coated onto 96-well plates and blocked with 1% FBS in PBS for 1 h at 37 °C. VLP-containing samples in blocking buffer were added, and bound antigen was detected with anti-DENV-2 MHIAF (1:5000) followed by HRP-conjugated goat antimouse IgG (1:6000; Leadgene, Taiwan). After incubation at 37 °C for 1 h, reactions were developed with TMB and stopped with 2 N H_2_SO_4_; absorbance at 450 nm was read using a MULTISKAN GO (Thermo Scientific, Waltham, MA)

### Cryo-EM and 3D Reconstruction

The structure of imD2VLPs was determined by single-particle cryo-EM. Purified samples (4 μL) were applied to glow-discharged Quantifoil holey carbon grids (Quantifoil GmbH, Germany). After 1 min in a high-humidity chamber, grids were blotted and plunge-frozen in liquid ethane using a Vitrobot Mark IV (FEI). Grids were kept in liquid nitrogen and transferred to a precooled cryo-transfer holder. Micrographs were recorded at a nominal magnification of 73,000× (pixel size 1.3785 Å) on a FEI Talos Arctica (Thermo Fisher Scientific) operated at 200 kV. Each exposure comprised 50 frames over 2.5 s, and frames were aligned in cryoSPARC.^51^ Micrographs underwent patch-CTF estimation, and 2D classification was performed to assess the heterogeneity and remove contaminants. An initial set of 45 particles was manually picked; the two most populated classes were used as templates for automated picking, yielding 12,563 particles from 350 micrographs. A second round of 2D classification removed 3512 nonspherical particles, leaving 9051 particles for *ab initio* model generation and homogeneous refinement (Figure S1). Global map resolution was estimated from the gold-standard Fourier shell correlation (FSC) between independently refined half-maps by using the 0.143 criterion. Gold-standard half-maps were generated internally in cryoSPARC by independent half-set refinement. The resulting FSC curves, as well as the half maps, the distribution of particle viewing directions used for the final reconstruction, were shown in the validation panel (Figure S7). Local resolution was estimated in cryoSPARC using the Local Resolution Estimation job on the consensus volume with the paired gold-standard half maps as input. To further test robustness, we performed focused C1 refinements using a soft 3D mask centered on the 5-fold axis and reconstructed independent focused half-maps.

### Plastic-Embedded Section Specimen

Pelleted cells were embedded in a Lowicryl/ethanol series with a gradually increasing ratio of Lowicryl/ethanol, followed by final infiltration in pure Lowicryl. Blocks were trimmed and ultrathinly sectioned to ∼90 nm using a diamond knife. Sections were collected on carbon-coated Formvar copper slot grids (FCF2010-Cu, EMS) stained with uranyl acetate and lead citrate and imaged on a JEOL JEM-1400 microscope.

### Coarse-Grained Model of imD2VLPs

The initial imD2VLP model was constructed by fitting the trimeric prM–E protein complex from the immature DENV-2 virion structure (PDB: 4B03)^52^ into the imD2VLP cryo-EM density map. Full-length complete atomistic models of E and prM proteins were constructed with sequences outlined in Experimental section-Plasmid and Cells. Modeler 9.10 was used to construct the homology models of the prM–E protein complex using immature DENV virion structure from PDB (PDB: 4B03). A single unit of the prM–E heterodimer structure was superimposed onto the 60 prM–E protein units of the initial full immature VLP model, composed of backbone atoms. The resulting atomistic immature VLP model was converted to a Martini 3.0 CG model with an elastic network (EN) to retain higher-order protein structure.^53^ We applied the EN to protein backbone beads using 0.5 to 0.9 nm cutoff distance and a force constant of 1000 kJ mol^–1^ nm^–2^. The EN was introduced only within each E protein domain (DI, DII, and DIII). In the case of the prM protein, the EN was used only on the structured portion of the protein (residues 1 to 80), while the TM regions of both E and prM proteins had no EN as described previously.^36^ We generated two lipid vesicles of ∼180 Å in agreement with the cryo-EM map (i.e., distance between center of lipid vesicle to the edge of inner leaflet) using the CHARMM-GUI Martini vesicle builder^54^ consisting of: (i) A phospholipid (PL) dominant composition, containing POPC (phosphatidylcholine 16:0/18:1): POPE (phosphatidylethanolamine 16:0/18:1): POPS (Phosphatidylserine 16:0/18:1) in a 60:30:10 ratio;^32,33^ (ii) A diacylglycerol-dominant composition, containing DG (diacylglycerol 18:0/20:4): SAPC (phosphatidylcholine 18:0/20:4): FA (fatty acid 18:1) in a 56:26:8 ratio in accordance with lipidomics data.^21^ In addition, for each lipid composition, two distinct vesicles with different total numbers of lipids were constructed. This was achieved by removing lipid molecules within either 1 (VLP_PL_-1 and VLP_DG_-1) or 1.5 Å (VLP_PL_-2 and VLP_DG_-2) octahedra of the protein CG beads. In total, four imD2VLP CG models were constructed. Each model was centered in a cubic box, and nonpolarizable MARTINI water was added together with 100 mM of NaCl to neutralize the overall system charge^55^ (Table S1). Each system energy was minimized for 50,000 steps using the steepest descent algorithm. Subsequently, the system was subjected to 400 ns equilibrium simulation in the NPT ensemble with position restraints applied to protein backbone beads with a force constant of 1000 kJ mol^–1^ nm^–2^. Bond lengths between backbone beads were constrained using the LINCS algorithm.^56^ Equations of motion were integrated using a leapfrog algorithm with a 10 fs time step. The Velocity rescale thermostat^57^ and Berendsen barostat^58^ were used to maintain temperature and pressure at 310 K and 1 bar, respectively. Three independent NPT ensemble unrestrained replicas were run for 2000–2500 ns each (Table S1). All CG simulations were run using Gromacs 2020.2 on 8 nodes containing 24 CPUs and 1 GPU each on the National Supercomputing Center (NSCC), Singapore.

### Targeted Molecular Dynamics (TMD) Simulations

TMD simulations were carried out using Gromacs 2018.2 ^59^ with Plumed 2.6.4 ^60,61^ by applying a harmonic potential to the root-mean-square deviation (RMSD) with respect to the target structure of protein backbone beads.^62,63^ Four independent force constants were used: 100, 500, 1000, and 10,000 kcal mol^–1^ Å^–2^ of the additional RMSD harmonic potential bias with respect to the target structure (Table S2). The RMSD harmonic potential was applied to E protein residues 1 to 495. In the case of the prM protein, steering forces were applied to the TM region alone (residues 150 to 166). Every 1000 steps of the TMD simulation, the RMSD between the current and target structure was calculated. The transition was linearly triggered over the simulation time using the RMSD difference between the immature and mature VLP states, totaling an RMSD of ∼58.7 Å. TMD was employed for 20,000,000 steps with a time step of 5 fs, resulting in a 100 ns trajectory for each system. All TMD simulations were carried out for the VLP-1 system with the PL dominant lipid vesicle (Table S1) and the same simulation settings were used as in the previous section. All TMD simulations were followed by 500 ns of unrestrained production simulations initiated from the final frame of each TMD trajectory to assess the stability of the steered configurations after removal of biasing forces.

### All-Atom Molecular Dynamics Simulations of Glycosylated Trimer Embedded in Lipid Bilayer

The coordinates for a single trimeric prM-E protein of the immature DENV (PDB entry 4B03) were used. Modeler 9.10 ^52^ was used to generate a homology model with E and M protein sequences as used in experiments. The lowest discrete optimized energy (DOPE) scored model structure with >97% amino acids in allowed regions of Ramachandran plot was used and embedded in a lipid bilayer composed of diacylglycerol (DG), phosphatidylcholine (PC) and fatty acid (FA) at 56:26:8 ratio as reported in Palur et al.^21^ Glycosylation sites N153 and N67 sites was modeled using the CHARMM-GUI glycan builder^64^ according to the glycan composition of DENV-2 reported in previous studies^44^ (Figure 7Dii). The default protonation states at neutral pH were assigned to the prM–E ionizable residue using the CHARMM36m force field.^65^ The trimeric unit was embedded into a lipid bilayer using the CHARMM-GUI membrane builder and solvated in a TIP3P water box^66,67^ plus 150 mM of NaCl, which also served to neutralize the overall charge of the system. The membrane embedded prM–E system was subjected to 5000 steps of steepest descent minimization followed by two and four steps of NVT and NPT equilibration steps, respectively. After each equilibration step the force constant on protein backbone and lipid headgroup atoms was gradually reduced from 4000 to 0 kJ mol^–1^ and 2000 to 0 kJ mol^–1^, respectively. A total of 1.5 ns of equilibration simulation using a 1 fs (NVT) and 2 fs (NPT) time step was run using the leapfrog algorithm. During equilibration, a 310 K temperature and 1 bar pressure were controlled using the Berendsen thermostat^58^ and semi-isotropic barostat. The Particle mesh Ewald (PME) method^68,69^ was used for long-range electrostatic interaction with a cutoff distance of 1.2 nm, and a switching function was applied after 1 nm. Production runs included Nosé-Hoover thermostat^70^ (303 K) and semi-isotropic Parrinello–Rahman barostat (1 bar)^71^ with 1 and 5 ps coupling constants, respectively. In the production runs, the positions of the TM backbone atoms of E and M protein were restrained using a force constant of 1000 kJ mol^–1^ nm^–2^. Restraints on the TM region were imposed to maintain the trimeric state of prM–E protein trimers, akin to the immature state. Three independent production simulations of 100 ns each were carried out with restraints on the TM region alone. All simulations employed Gromacs 2020.2 on 4 nodes with 24 CPUs and 1 GPU on the National Supercomputing Center (NSCC), Singapore.

### Simulation Analysis

To quantify the structural changes observed during TMD simulations driving the imD2VLP to mD2VLP transition, we measured three specific angles between either E protein monomers or dimers: ⊖, Φ, and δ (Figure 6D–F). We defined ⊖ as the angle between vectors defined by E protein monomers, as shown in Figure 6D, where each vector connects the center of mass (COM) of DII and COM of DIII of a respective E protein monomer. Φ was defined as the angle between two vectors, where the first vector is defined by the COM of DII and DIII of an E monomer, and the second corresponds to COM of the entire VLP and COM of DIII of the respective E protein monomer (Figure 6E). δ was defined as the angle between the vector connecting the COM of DIII and DII of each monomer at time *t* = 0 and the same vector at different time points during the TMD-1 simulation (Figure 6F). Similarly, SHTM-specific angles i.e., α1 (angle between residues 421, 451, and 471) and α2 (angle between residues 396, 421, and 451) were defined and measured to capture the structural changes (Table S4). Importantly, we utilized the mature DENV VLP CG-simulations trajectory to calculate the above structural features from our previous study^21,36^ to compare with unrestrained immature and TMD simulations from the current study. In all simulations, Gromacs tools were used to perform PCA, calculate RMSDs, and calculate the number of contacts and angles, while VMD, PyMOL, and ChimeraX were used to visualize molecules and simulation trajectories. Lipid escape and protrusion were quantified for each coarse-grained molecular dynamics (CG-MD) trajectory using lipid headgroup (PO4) bead positions (Figure S6). For each simulation frame, the vesicle center of mass (COM) was calculated from all lipid headgroup beads, and the radial distances of individual headgroups were measured relative to this center. The vesicle surface radius was defined as the median radial distance of all of the lipid headgroup beads. A lipid was classified as protruding or escaped if its PO4 bead was located more than 5.0 nm beyond this vesicle surface radius. To enable comparison across lipid species with different abundances, lipid escape was additionally quantified as a normalized escape fraction, defined as the number of escaped lipids of a given species divided by the total number of lipid species present in the vesicle (POPC, POPE, or POPS). This normalization accounts for differences in lipid composition (POPC:POPE:POPS ≈ 6:3:1) and allows a direct comparison of escape propensity across lipid types. The vesicle radius was quantified as the median radial distance of lipid headgroup (PO4) beads from the COM, reporting global vesicle expansion or compression over time. Vesicle sphericity was quantified by using a radial-distribution-based metric that compares the dispersion of lipid headgroup radial distances to their mean value. Values approaching unity indicate a near-spherical vesicle, whereas lower values reflect increasing surface roughness or global deformation (Figure S6). Two-dimensional angular maps of vesicle deformation were generated by projecting lipid headgroup positions onto spherical angular coordinates (⊖, φ)­relative to the COM (Figure S8). For each angular bin, the local mean radial position of lipid headgroups was computed and compared to the global mean vesicle radius. The resulting radial displacement reports whether the vesicle surface at a given angular location protrudes outward or is locally indented relative to the average vesicle surface. Positive values indicate outward protrusions, whereas negative values indicate inward deformations. Two-dimensional lipid enrichment was quantified by computing the local surface density of each lipid species within each angular bin and normalizing it by the expected density under uniform random mixing. Enrichment ratios greater than unity indicate preferential localization of a lipid species in each angular region, whereas values below unity indicate depletion. This analysis identifies lipid sorting and spatial organization on the vesicle surface. All angular maps were time-averaged over the corresponding simulation trajectories and smoothed in angular space using a Gaussian filter to reduce statistical noise while preserving large-scale spatial patterns.

## Supplementary Material









## Data Availability

The EM structures have been deposited in the Electron Microscopy Data Bank (EMDB ID code: 67800).

## Notes

Figure S1: Representative 2D class averages from reference-free classification; Figure S2: Root mean square fluctuation (RMSF) and solvent accessible surface area (SASA) of VLP_PL_-1 and VLP_PL_-2 systems; Figure S3: Principal component analysis (PCA), simulation-generated VLP densities and pr-E interactions in VLP_PL_-1 and VLP_PL_-2 systems; Figure S4: Stem-helix transmembrane (SHTM) region – lipid interactions and solvent accessibility of E protein domains; Figure S5: Maturation pathway analysis of VLPs using targeted MD (TMD) simulations; Figure S6: Structural and morphological stability of VLP models; Figure S7: Global map validation and focused C1 analysis of membrane- and glycan- associated features in the imD2VLP reconstruction; Figure S8: Angular maps of lipid-type–specific membrane deformation and lipid enrichment relative to embedded protein helices in VLP_PL_-1and VLP_PL_-2vesicles; Figure S9: Time-resolved lipid protrusion and escape dynamics during coarse-grained molecular dynamics simulations of immature DENV-2 VLPs; Figure S10: Structural arrangement of E and pr proteins in virion and VLP; Table S1: Immature dengue serotype 2 virus-like particles (imD2VLPs) CG model simulation setup; Table S2: All atom glycosylated prM–E trimeric protein simulation setup; Table S3: Initial (immature VLP) and final (mature VLP) φ, δ and ⊖ angle values from the TMD simulations; Table S4: Comparison of immature and mature VLP and virion angles (PDF)
Movie 1: Atomistic model of immature VLP particle fitted into to the cryo-EM density map, displayed in a 360° rotation (MP4)
Movie 2: Immature to mature VLP transition pathway over the course of the targeted molecular dynamics simulation (TMD-1) (MPG)
Movie 3: All atom simulation of prM–E glycosylated trimeric protein embedded in DG dominant lipid bilayer (MPG)
