# Supplementary material for: Multiscale Simulations and Cryo-Electron Microscopy Reveal the Transition Pathway of Dengue Virus-like Particle Nanoassembly
Source: ACS Nano. 2026 Feb 16;20(8):6845–63. doi: 10.1021/acsnano.5c17047 (PMC12961931; doi:10.1021/acsnano.5c17047)
Supplement: Supplementary file 1 [file nn5c17047_si_001.pdf]

Supporting Information

Multiscale simulations and Cryo-Electron Microscopy Reveal Transition Pathway of Dengue Virus–Like Particle Nanoassembly

Venkata Raghuvamsi Palur[2#], Guan-Wen Chen[1#], Day-Yu Chao[3], Wen-Shuo Kuo[7], Ya-Na Wu[1], Jedhan U. Galula[3], Chun-Hsiung Wang[5], Fan-Chi Chen[6], Peter J. Bond[2,4], Jan K. Marzinek[2,*] & Shang-Rung Wu[1,*]

[1] School of Dentistry & Institute of Oral Medicine, College of Medicine, National Cheng Kung University, Tainan 70101, Taiwan

[2] Bioinformatics Institute (BII), Agency for Science, Technology and Research (A*STAR), 30 Biopolis Street, #07-01 Matrix, Singapore 138671, Republic of Singapore

[3] Graduate Institute of Microbiology and Public Health, College of Veterinary Medicine, National Chung Hsing University, Taichung 40227, Taiwan

[4] Department of Biological Sciences, National University of Singapore, Singapore, Republic of Singapore

[5] Institute of Biological Chemistry, Academia Sinica, Taipei 11529, Taiwan

[6] Microbial Genomics PhD Program, National Chung Hsing University and Academia Sinica, Taichung 40227, Taiwan

[7] Center for Allergy Immunology and Microbiome (AIM), China Medical University Children's Hospital/China Medical University Hospital, China Medical University, Taichung 40447, Taiwan

* Correspondence: marzinekj@a-star.edu.sg and z10208056@ncku.edu.tw

#These authors contributed equally to this work.

Legends

Movie1: Atomistic model of immature VLP particle fitted into to the cryo-EM density map, displayed in a 360° rotation. E-pr proteins are shown in cartoon representation with each monomer displayed in a distinct color. Density map is shown as grey transparent surface representation.

Movie2: Immature to mature VLP transition pathway over the course of the targeted molecular dynamics simulation (TMD-1). E-pr proteins and lipids are shown as spheres: each monomer is displayed in a distinct color, lipids tails are shown in cyan while lipid headgroups in purple, blue and brown.

Movie3: All atom simulation of E-prM glycosylated trimeric protein embedded in DG dominant lipid bilayer. Protein is shown in cartoon representation with each monomer displayed in a distinct color. Glycans at N67 and N153 are shown as sticks and spheres (cyan – carbon, red – oxygen, blue – nitrogen, white – hydrogen). Lipid bilayer is shown as black spheres.

Table S1. Immature dengue serotype 2 virus-like particles (imD2VLPs) CG model simulation setup.

System	Box Size (nm)	Total number of lipids	Lipid membrane composition	NaCl concentration	Production run (ns)
VLP_{PL}-1	41.5×41.5×41.5	~2,000	POPC:POPE:POPS (60:30:10)	100mM	3 × 2500 ns
VLP_{PL}-2		~1,000			
VLP_{DG}-1	41.5×41.5×41.5	~2,000	DG:SAPC:FA (56:26:8)		3 × 2000 ns
VLP_{DG}-2		~1,000			

Table S2. All atom glycosylated E-prM trimeric protein simulation setup.

Box Size (nm)	No. of water molecules	Lipid membrane composition	NaCl concentration	Production run (ns)
15.5×15.5×18.3	100,536	DG:SAPC:FA (56:26:8)	100mM	3 × 100

Table S3. Initial (immature VLP) and final (mature VLP) φ, δ and ϴ angle values from the TMD simulations. Values represent averages across all 60 monomers, and error bars indicate standard deviations.

System	Simulation	Force constant (kcal mol^{-1} $Å^{-2}$)	Φ_{av} (t=0)	Φ_{av} (t=100ns)	Θ_{av} (t=0)	Θ_{av} (t=100ns)	δ_{av} (t=0)	δ_{av} (t=100ns)
**Immature VLP-1**	**TMD-1**	100	98.28±0.17	82.08±2.3	142.3±0.23	147.7±3.1	1.61±0.7	64.6±5.0
	**TMD-2**	500		76.22±1.4		156.6±1.8		67.0±5.9
	**TMD-3**	1,000		75.09±0.9		158.3±1.1		67.8±7.3
	**TMD-4**	10,000		74.30±0.6		160.1±0.8		68.3±4.2
**Mature VLP-1**	**-**	-	-	106.0±0.7	-	160.9±0.9	-	-

Table S4. Comparison of immature and mature VLP and virion angles: ϕ, ϴ, $\alpha 1$ and $\alpha 2$ angles from our current and previous CG structural models of DENV. The values were averaged across all 60 (DENV VLP) or 180 (DENV virion) monomers (av) and error bars correspond to standard deviations. Difference (Δ) between initial and final angles were calculated as absolute values with error bars corresponding to standard propagation of the individual errors.

System	Φ_{av} (º)	|Δ Φ| (º)	Θ_{av} (º)	|Δ ϴ | (º)	$\alpha 1$ (º)	$\alpha 2$ (º)
**Immature VLP**	98.3 ± 0.2	7.7 ± 0.7	142.3 ± 0.2	18.6 ± 0.9	72.2 ± 2.6	37.1 ± 2.6
**Mature VLP**	106.0 ± 0.7		160.9 ± 0.9		82.2 ± 2.5	80.1 ± 1.5
**Immature virion**	100.4 ± 0.6	18.4 ± 2.3	111.9 ± 0.01	53.0 ± 0.02	70.9 ± 0.1	33.4 ± 0.1
**Mature virion**	82.0 ± 2.2		164.9 ± 0.02		67.0 ± 0.1	71.0 ± 0.1

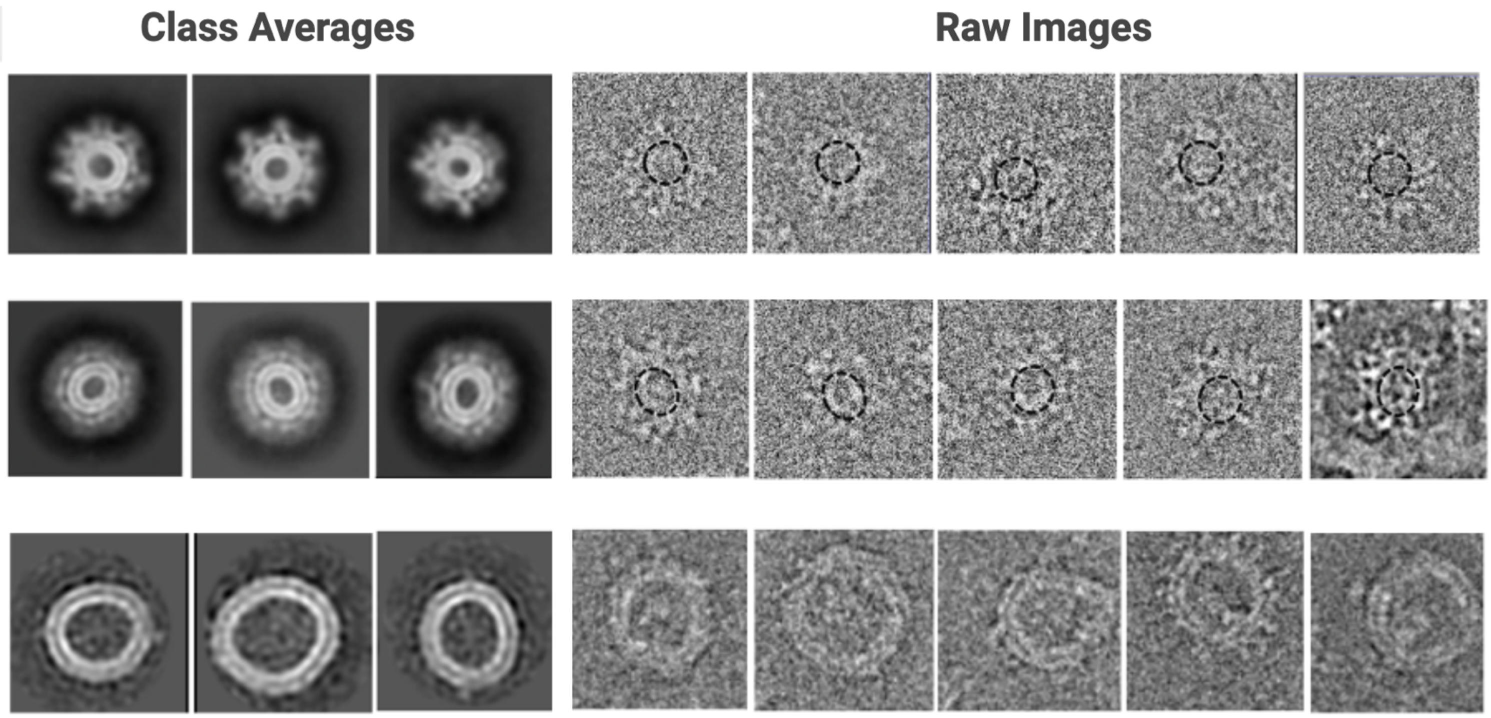

Figure S1. Representative 2D class averages from reference-free classification. The left panel listed the class averages while the right panel lists the raw images.

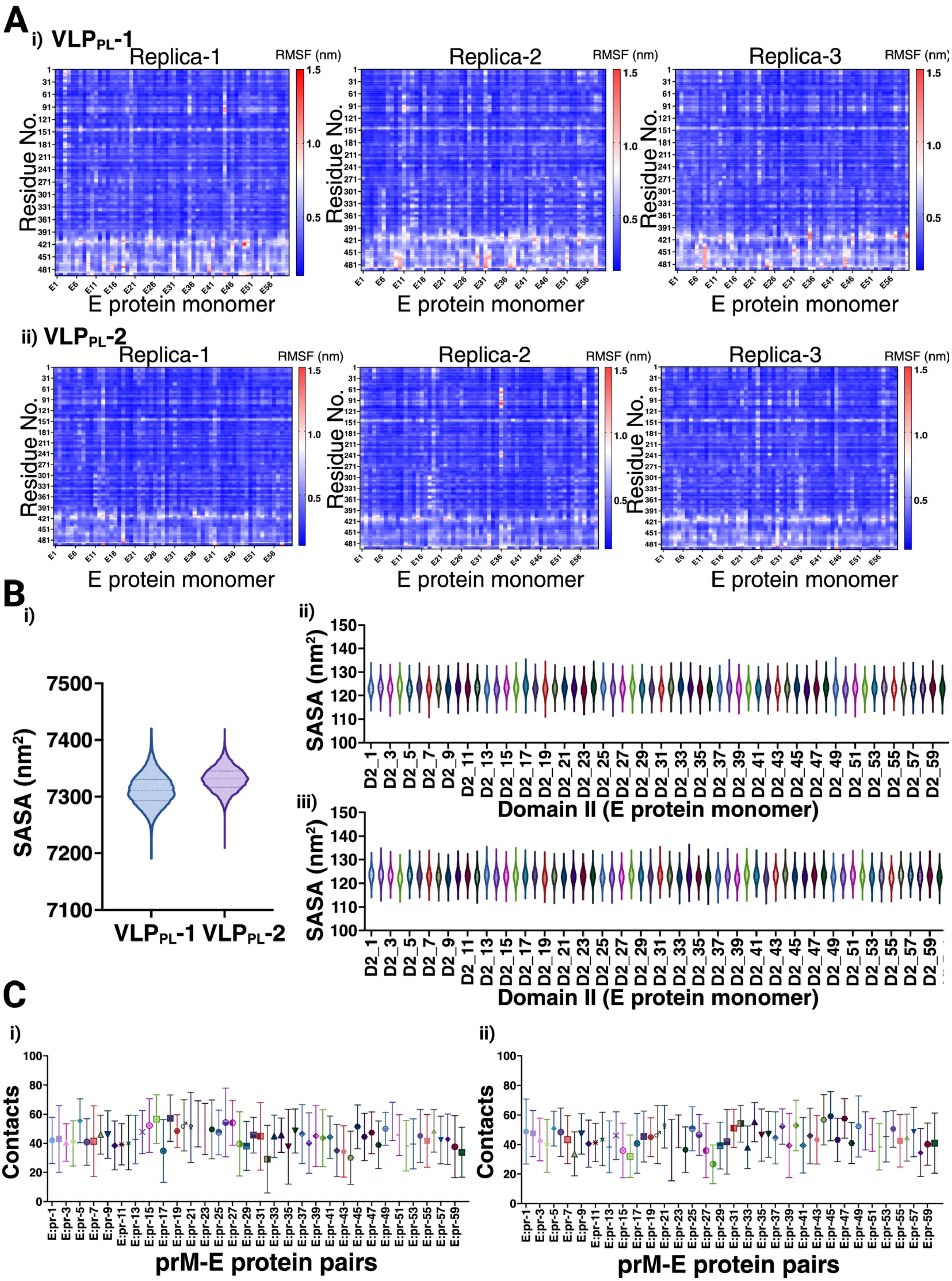

A
i) VLP$_{PL}$-1
Replica-1
Replica-2
Replica-3
RMSF (nm)
Residue No.
E protein monomer
ii) VLP$_{PL}$-2
Replica-1
Replica-2
Replica-3
RMSF (nm)
Residue No.
E protein monomer
B
i)
SASA (nm^2)
VLP$_{PL}$-1
VLP$_{PL}$-2
ii)
SASA (nm^2)
Domain II (E protein monomer)
iii)
SASA (nm^2)
Domain II (E protein monomer)
C
i)
Contacts
prM-E protein pairs
ii)
Contacts
prM-E protein pairs

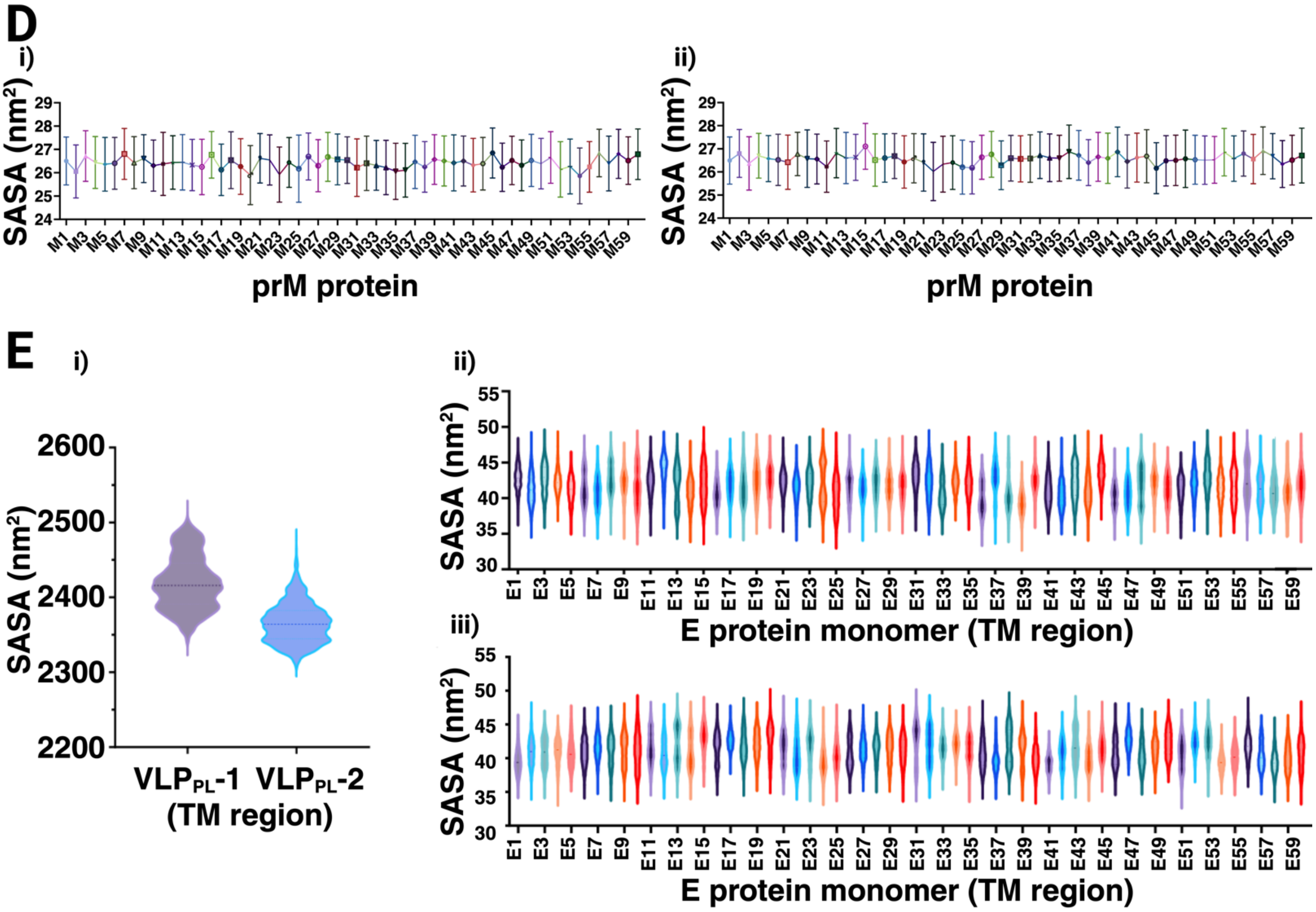

Figure S2. Root mean square fluctuation (RMSF) and solvent accessible surface area (SASA) of VLP_{PL}-1 and VLP_{PL}-2 systems. **(A)** Heatmap showing per-residue RMSF values of each E protein averaged across three replicas for each protein monomer. **(B)** Violin plot of the combined VLP SASA of DII of all 60 monomers (i), together with each E protein monomer DII from VLP_{PL}-1 (ii) and VLP_{PL}-2 (iii). **(C)** Average number of contacts for each monomer between pr and DII for VLP_{PL}-1 (i) and VLP_{PL}-2 (ii) systems. **(D)** Average SASA value for each pr domain from VLP_{PL}-1 (i) and VLP_{PL}-2 (ii). **(E)** Violin plot of the SASA of TM of all 60 monomers (i) as well as individual TM SASA from each E protein for VLP_{PL}-1 (ii) and VLP_{PL}-2 (iii). All data were calculated across three replicas. Error bars corresponded to the standard deviation.

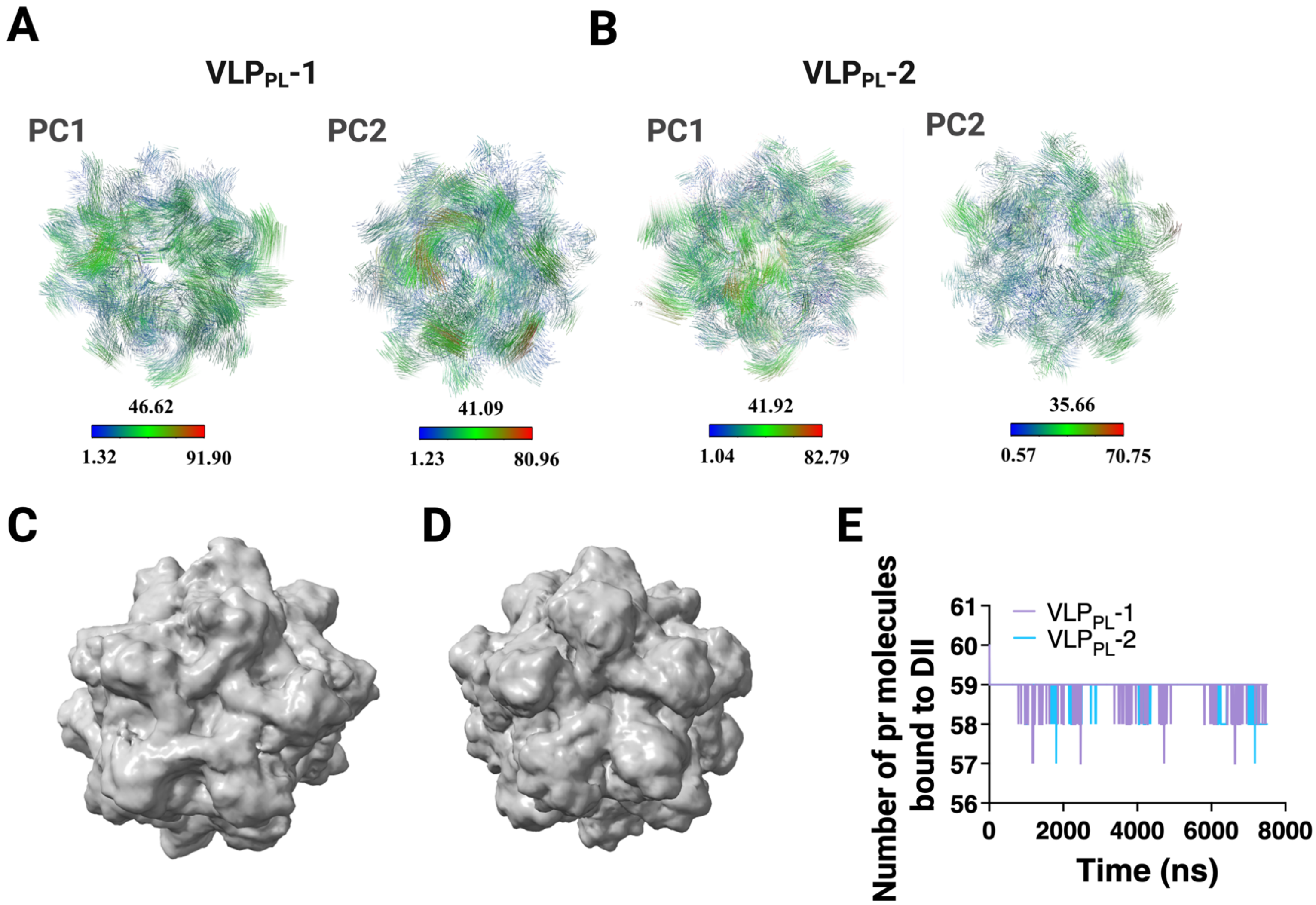

Figure S3. **Principal component analysis (PCA), simulation-generated VLP densities and pr-E interactions in VLP$_{PL}$-1 and VLP$_{PL}$-2 systems. (A-B)** Porcupine plots show the most dominant motions represented by the first and second principal components (PC1 and PC2) from the PL-dominant lipid system. PCA is extracted from a combined 7,500 ns-long simulation trajectory. PC1 accounts for ~41% to ~45% of the total variance for VLP$_{PL}$-1 and VLP$_{PL}$-2, respectively. PCA was performed on every third protein backbone bead of each VLP trajectory. **(C-D)** Surface representations of density maps generated from simulation trajectories for VLP$_{PL}$-1 **(C)** and VLP$_{PL}$-2 **(D)** systems. The VMD VolMap tool was used to generate the densities at a resolution of 5Å. **(E)** The number of pr molecules within 0.6 nm of DII for all 60 prM-E protein monomers is shown over simulation time.

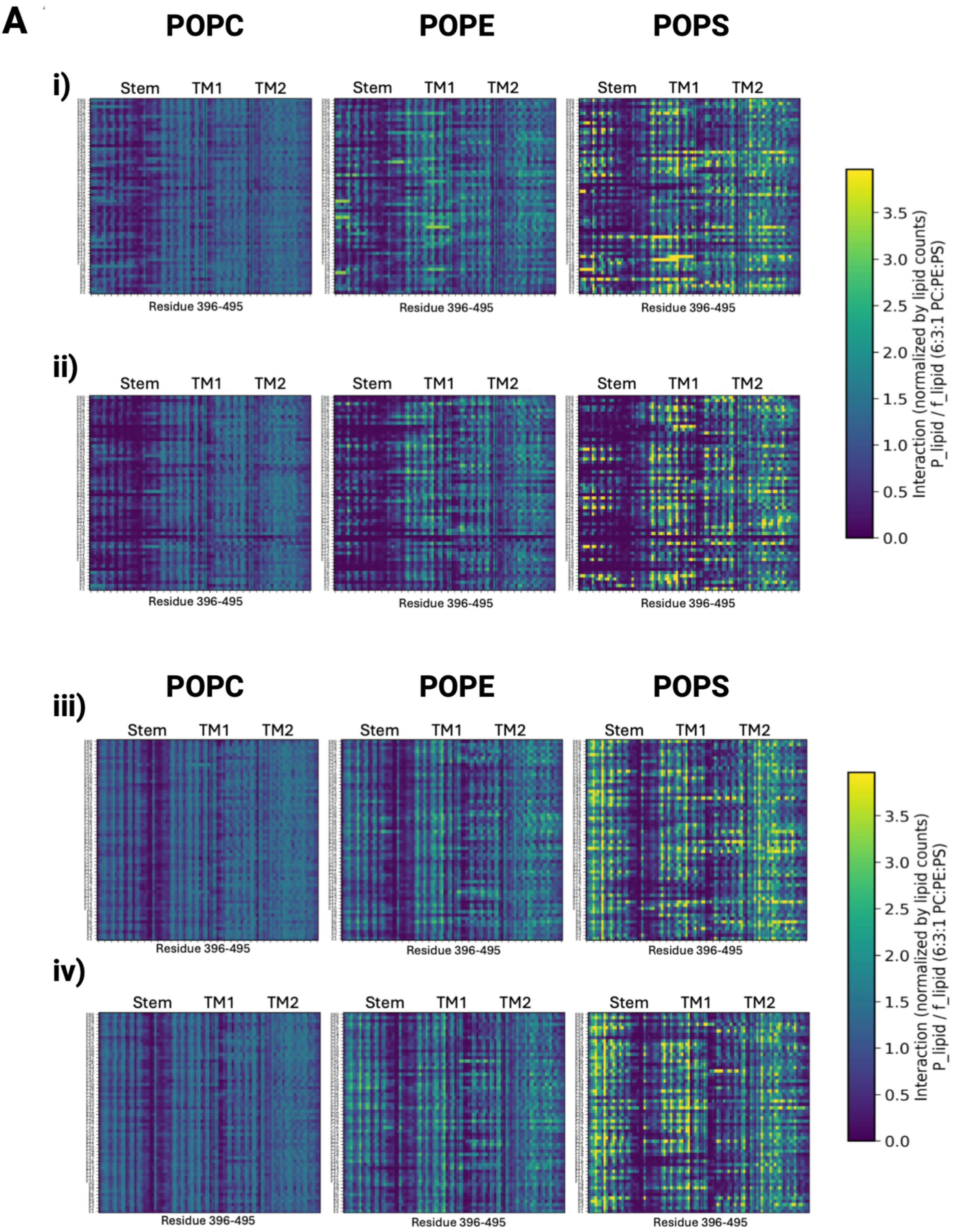
A
POPC
POPE
POPS
i)
Stem
TM1
TM2
Residue 396-495
ii)
iii)
iv)
3.5
3.0
2.5
2.0
1.5
1.0
0.5
0.0
Interaction (normalized by lipid counts)
P_lipid / f_lipid (6:3:1 PC:PE:PS)

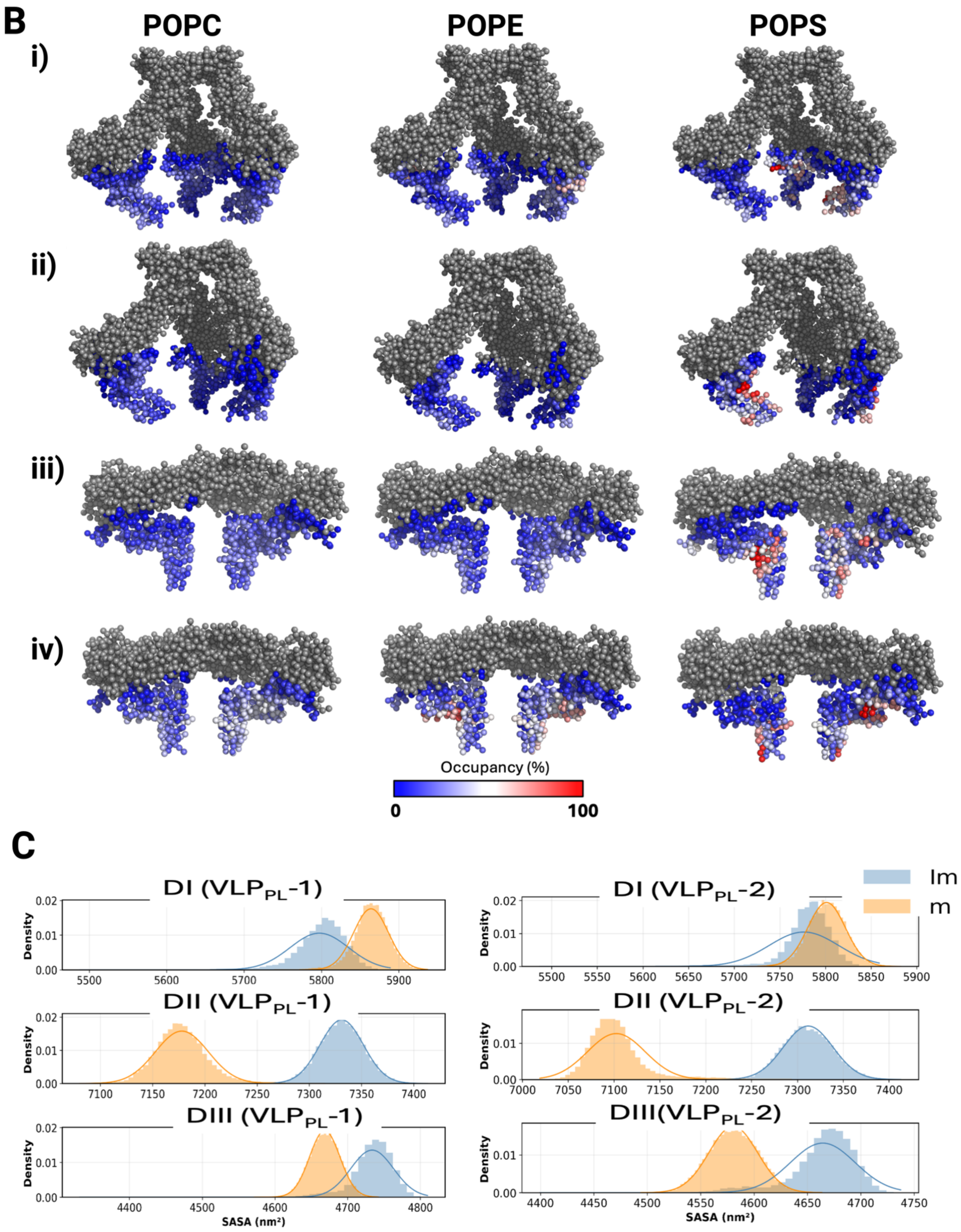
B
POPC
POPE
POPS
i)
ii)
iii)
iv)
Occupancy (%)
0
100
C
DI (VLP$_{PL}$-1)
DI (VLP$_{PL}$-2)
DII (VLP$_{PL}$-1)
DII (VLP$_{PL}$-2)
DIII (VLP$_{PL}$-1)
DIII(VLP$_{PL}$-2)
Im
m
Density
SASA (nm²)

Figure S4. Stem-helix transmembrane (SHTM) region – lipid interactions and solvent accessibility of E protein domains in mature and immature DENV VLPs. (A)-(B) Per-residue lipid occupancy between SHTM region and POPC, POPE and POPS lipids calculated from combined triplicate CG MD simulation trajectories for: immature (i) $^{Im}VLP_{PL}$-1, (ii) $^{Im}VLP_{PL}$-2 and mature: (iii) $^{m}VLP_{PL}$-1, (iv) $^{m}VLP_{PL}$-2 VLP constructs. In **(A)**, per-residue occupancies were shown as heat maps for each of the 60 E proteins (Y axis) and were calculated as the fraction of frames in which a lipid molecule was observed within 0.6 nm of the SHTM region, followed by normalization with respect to the number of lipids. In **(B)**, the occupancies were calculated as the fraction of frames in which a lipid molecule was observed within 0.6 nm of the SHTM region. Protein residues are shown as spheres and colored based on the occupancy from 0 to 100% (blue – white – red). **(C)** Distribution of the SASA of E protein domains: DI, DII and DIII from the combined triplicate simulation trajectory of mature (m) and immature (Im) CG models.

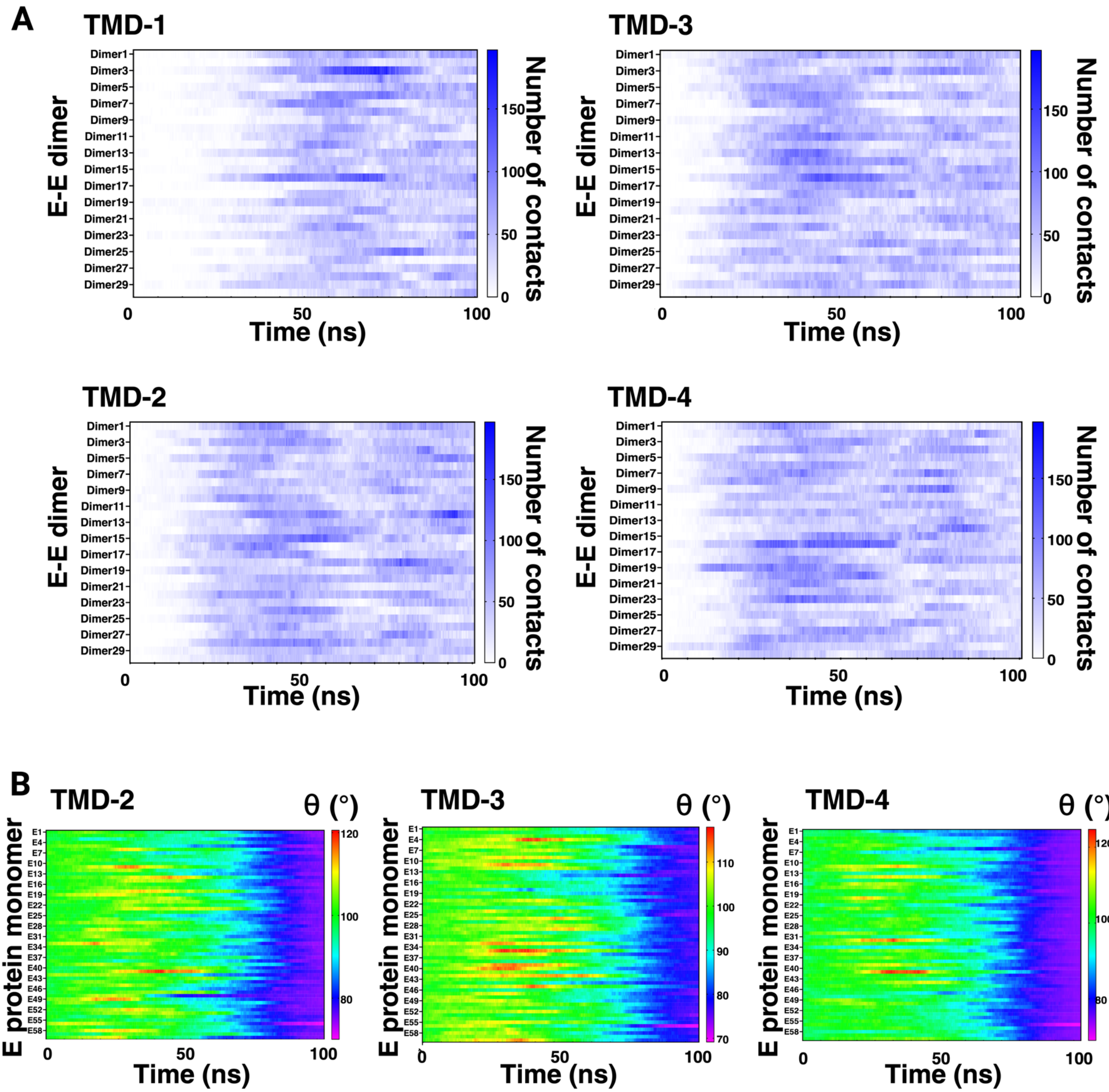
A
TMD-1
TMD-3
TMD-2
TMD-4
E-E dimer
Dimer1
Dimer3
Dimer5
Dimer7
Dimer9
Dimer11
Dimer13
Dimer15
Dimer17
Dimer19
Dimer21
Dimer23
Dimer25
Dimer27
Dimer29
Number of contacts
0
50
100
150
Time (ns)
B
θ (°)
E protein monomer
E1
E4
E7
E10
E13
E16
E19
E22
E25
E28
E31
E34
E37
E40
E43
E46
E49
E52
E55
E58
70
80
90
100
110
120

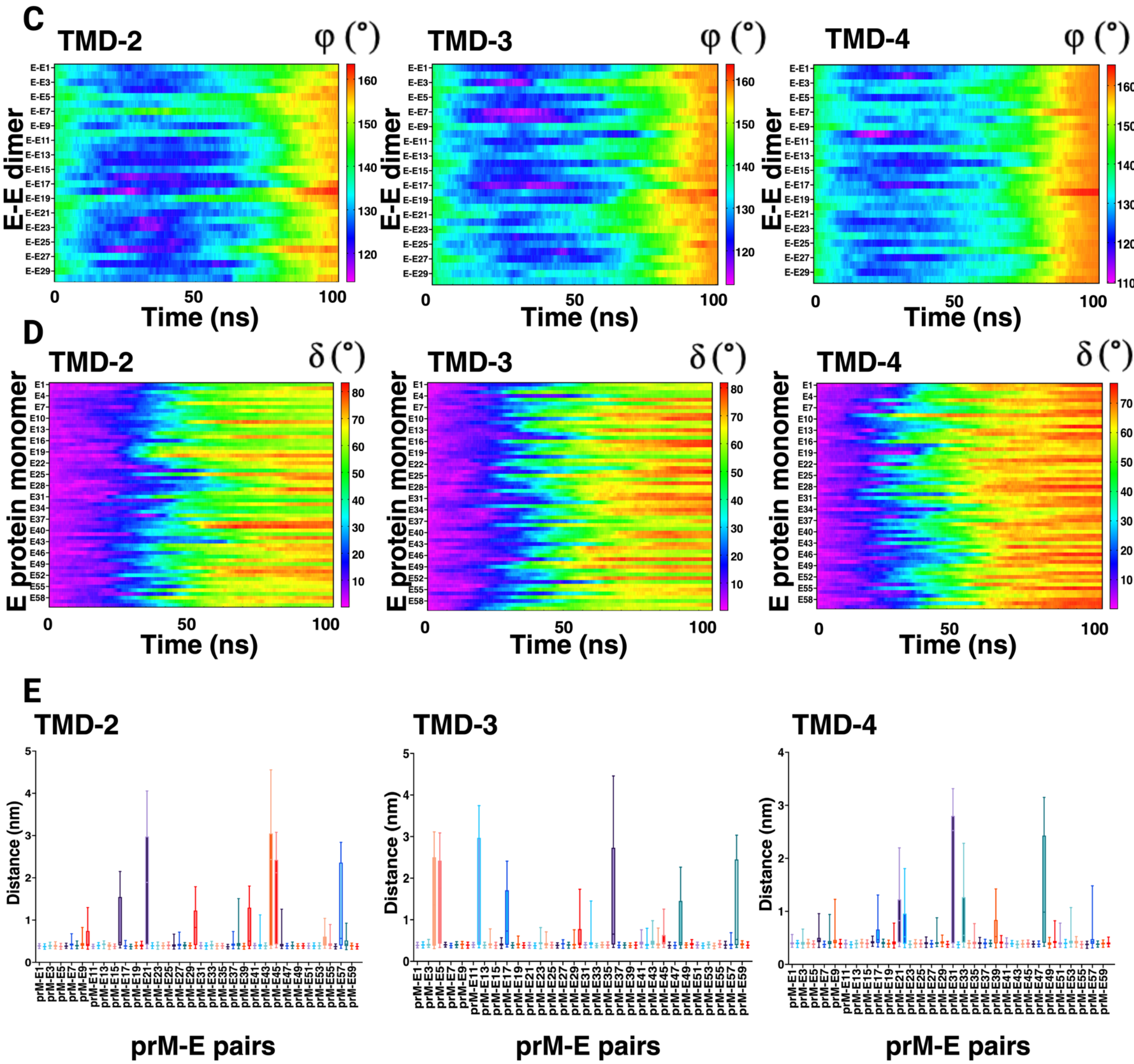

Figure S5. Maturation pathway analysis of VLPs using targeted MD (TMD) simulations. (A) Heatmap shows monomer-monomer contacts for each of 30 E protein dimers over the course of each TMD simulation. **(B)** Heatmap shows the changes in angle Θ between E protein dimers over the course of each TMD simulation. **(C)** Heatmap shows the changes in angle ϕ over the course of each TMD simulation. **(D)** Heatmap shows the changes in angle δ over the course of each TMD simulation. **(E)** Box plots show the distributions of distances between domain II and pr molecules for each of 30 E-prM proteins, averaged over the course of each TMD simulation.

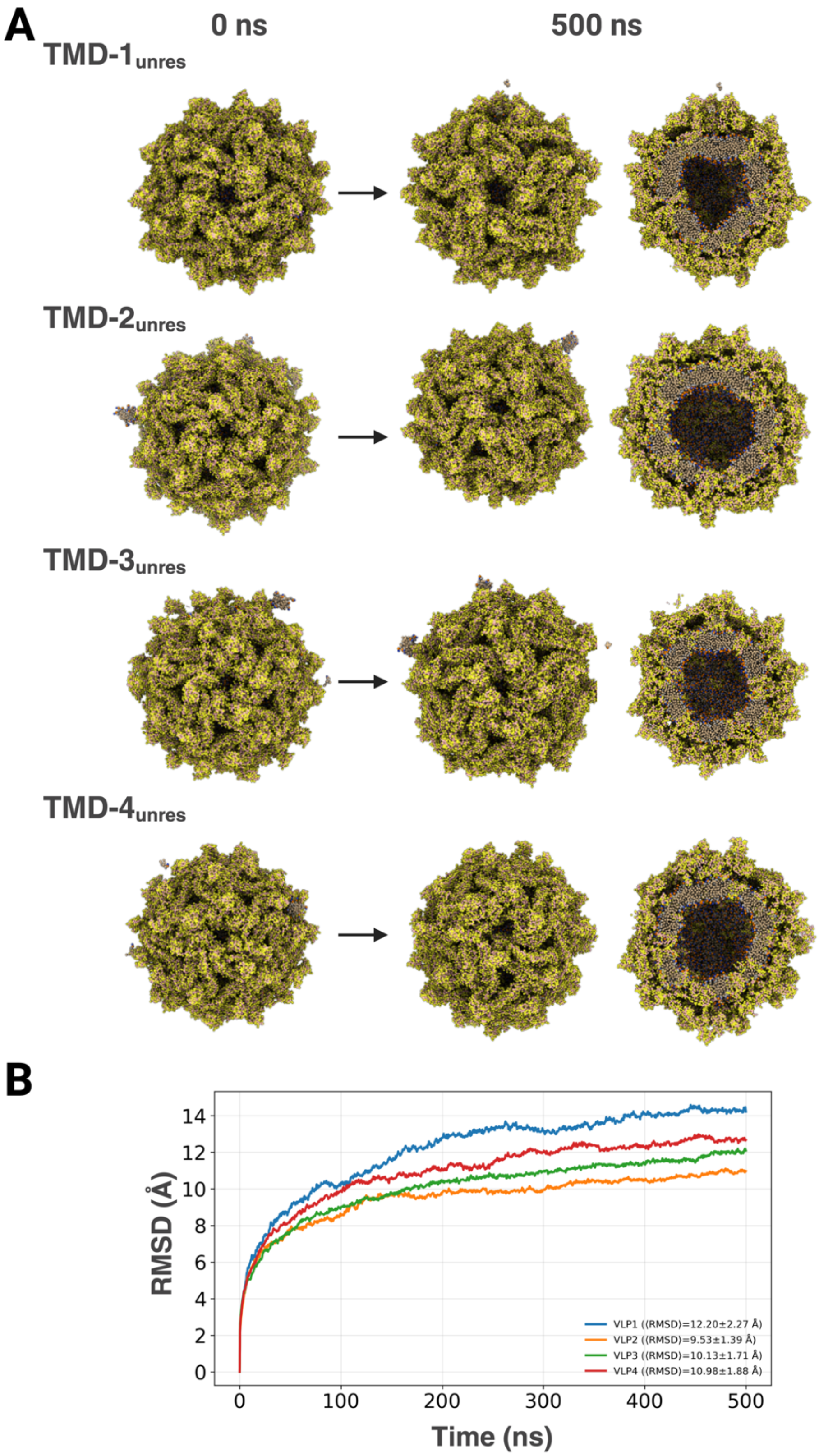
A
0 ns
500 ns
TMD-1unres
TMD-2unres
TMD-3unres
TMD-4unres
B
RMSD (Å)
Time (ns)
VLP1 ((RMSD)=12.20±2.27 Å)
VLP2 ((RMSD)=9.53±1.39 Å)
VLP3 ((RMSD)=10.13±1.71 Å)
VLP4 ((RMSD)=10.98±1.88 Å)

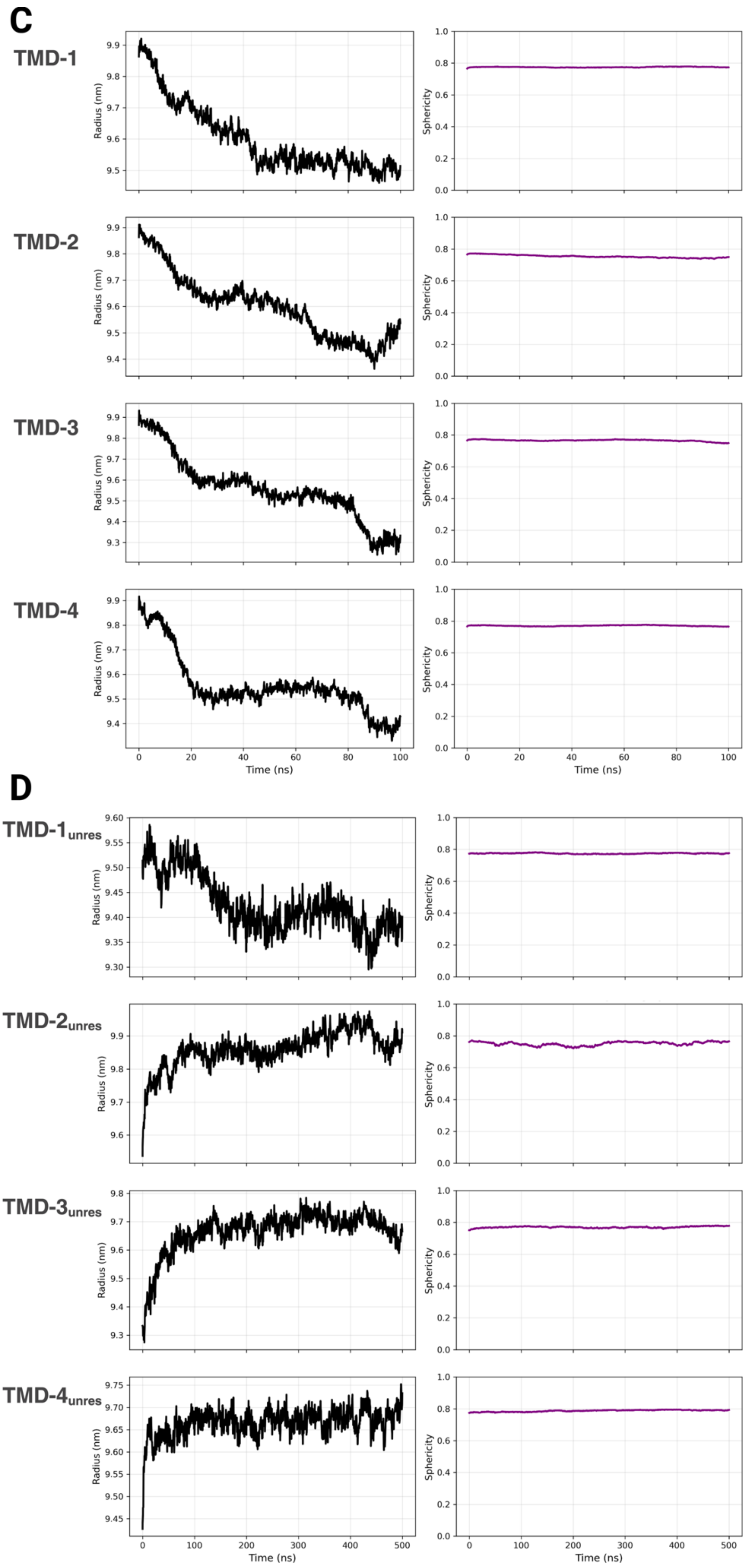

C
TMD-1
TMD-2
TMD-3
TMD-4
Radius (nm)
Sphericity
Time (ns)
D
TMD-1unres
TMD-2unres
TMD-3unres
TMD-4unres
Radius (nm)
Sphericity
Time (ns)

Figure S6. Structural and morphological stability of VLP models during extended unrestrained simulations following TMD-driven maturation transitions. **(A)** Simulation snapshots at the initial time (0 ns) and at the final time (500 ns) for unrestrained post-TMD simulations. **(B)** Root-mean-square deviation (RMSD) as a function of simulation time. **(C–D)** Time evolution of the vesicle radius and sphericity of the lipid envelope from TMD trajectories (**C**) and from unrestrained simulations initiated from the final frame of each TMD simulation (**D**). The vesicle radius (left panels) is defined as the median radial distance of lipid headgroup (PO4) beads from the vesicle center of mass and reports global vesicle expansion or compression over time. The vesicle sphericity (right panels) is quantified using a radial-distribution-based metric, $\Psi = 1 - \sigma_r/\langle r \rangle$, where $\langle r \rangle$ and σ_r denote the mean and standard deviation of lipid headgroup radial distances, respectively. Values approaching unity indicate a near-spherical vesicle, while lower values reflect increasing surface roughness or global deformation.

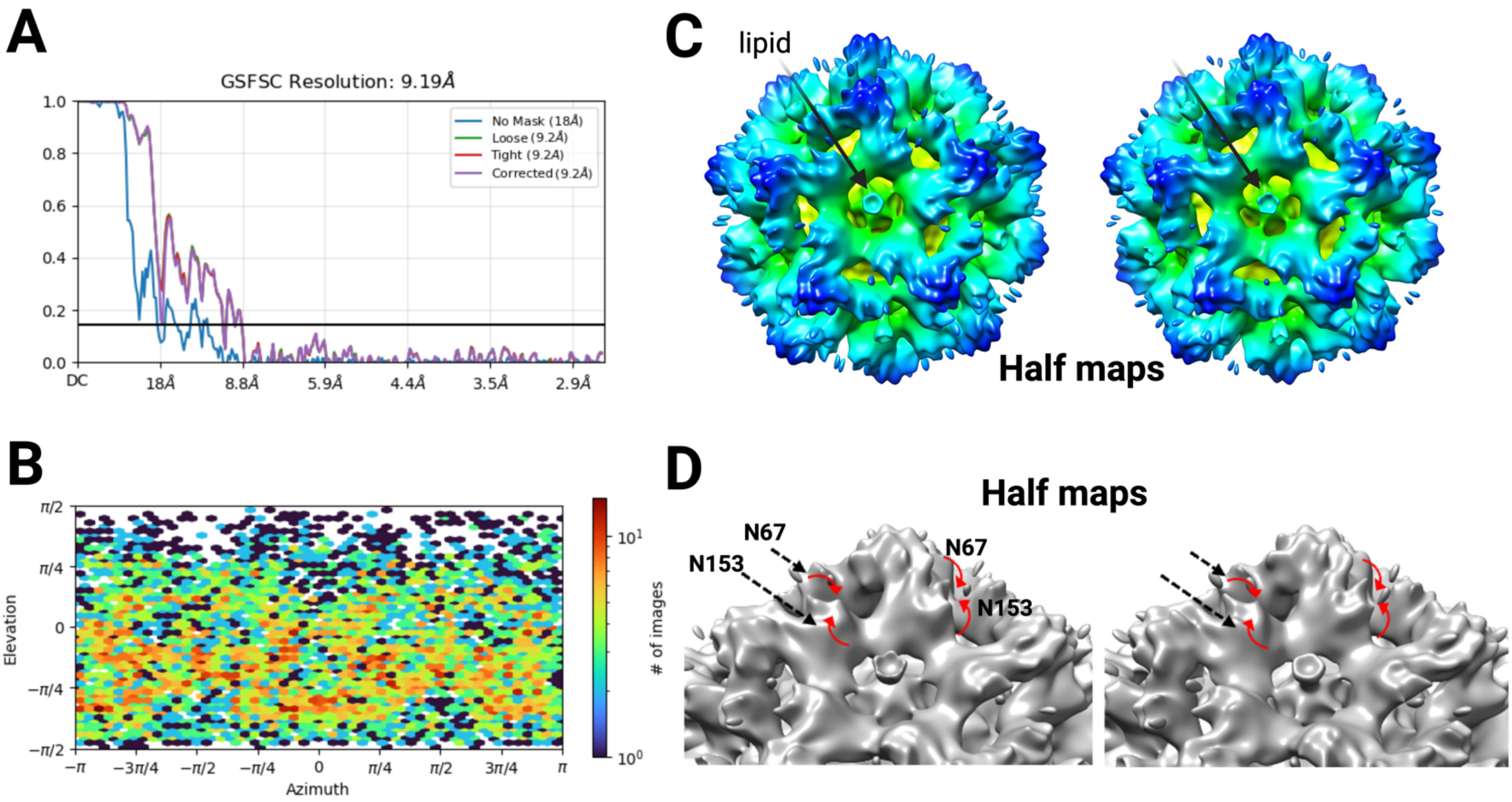

Figure S7. Global map validation and focused C1 analysis of membrane- and glycan-associated features in the imD2VLP reconstruction. (**A**) Gold-standard Fourier shell correlation (GSFSC) between independently refined half-maps with no mask (blue) and with loose, tight and phase-randomization–corrected soft masks (green, red, magenta) is shown. The 0.143 criterion indicates a global resolution of 9.2 Å for the masked map. (**B**) The angular distribution of particle viewing directions used for the final reconstruction is shown, plotted as a 2D histogram of elevation versus azimuth; the color scale indicates the number of particles per bin. (**C**) Focused C1 half-map reconstructions using a soft mask centered on the five-fold axis, showing reproducible membrane-proximal density at the inner leaflet near the axis (arrows) in both half-maps, consistent with a variably occupied lipid-associated feature. (**D**) Focused C1 half-maps using a mask encompassing the neighborhood of the N67 and N153 sites on E, revealing reproducible site-adjacent density (arrows) in both halves, consistent with conformationally dynamic, glycan-compatible features.

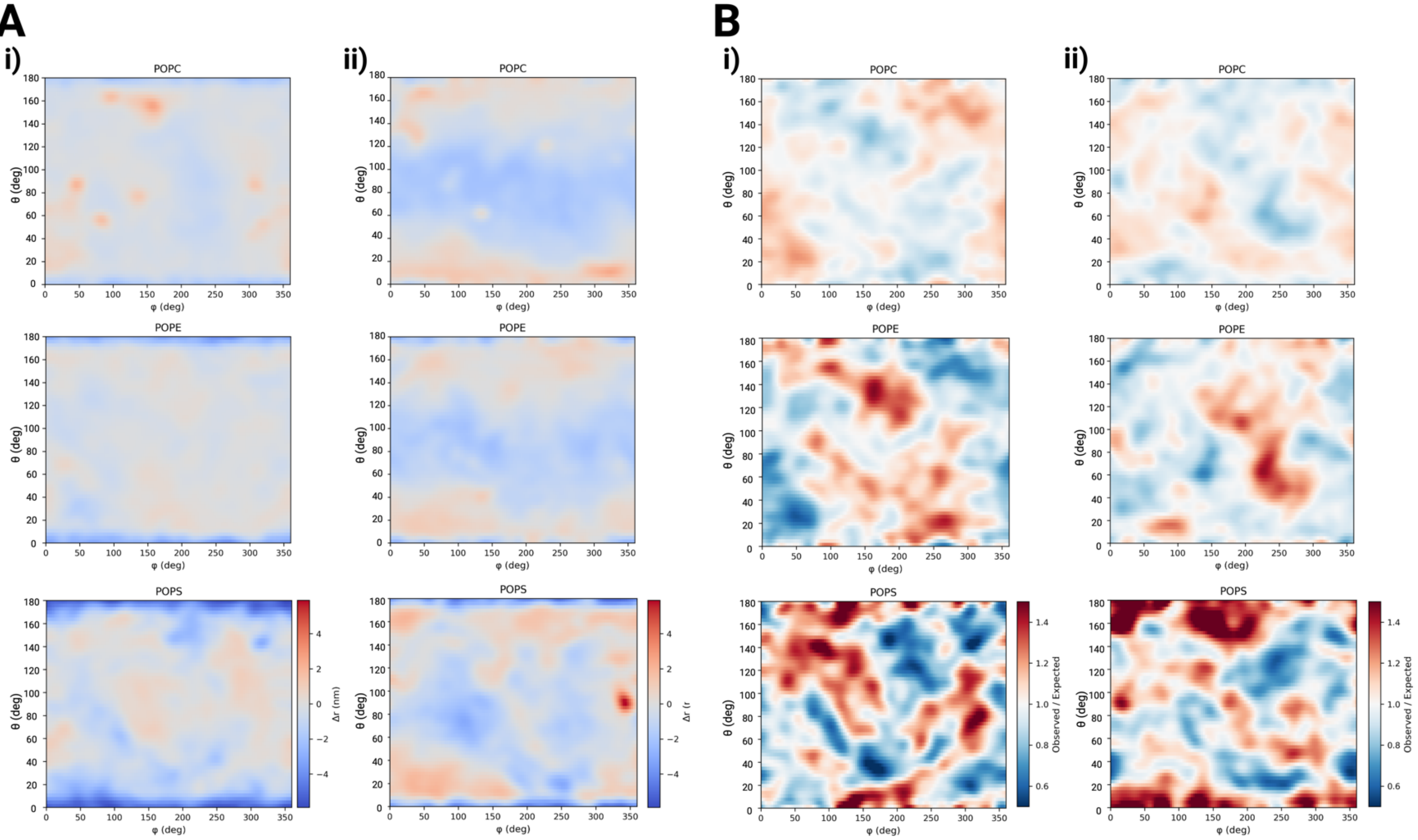

Figure S8. Angular maps of lipid-type–specific membrane deformation and lipid enrichment relative to embedded protein helices in VLP$_{PL}$-1 and VLP$_{PL}$-2 vesicles. (A) Two-dimensional angular maps showing the lipid-type–specific radial displacement profiles of the lipid envelope, calculated from combined triplicate unrestrained simulation trajectories of VLP$_{PL}$-1 (i) and VLP$_{PL}$-2 (ii). Lipid headgroup positions were referenced to the instantaneous vesicle center of mass and projected into spherical angular coordinates (θ, ϕ). For each angular bin, time-averaged quantities were calculated and smoothed in angular space using a Gaussian filter. Radial displacement maps report the local deviation of the lipid-specific mean radial position from the global mean vesicle radius ($\Delta r = r_{local} - \langle r \rangle$), where positive values indicate outward protrusion and negative values indicate inward deformation of the vesicle surface. **(B)** Two-dimensional angular maps of lipid organization in the vesicle envelope of VLP$_{PL}$-1 (i) and VLP$_{PL}$-2 (ii) calculated from the same trajectories. Lipid enrichment maps report the ratio of observed lipid density to the expected density under uniform random mixing (Observed/Expected), with values greater than unity indicating enrichment and values below unity indicating depletion. Black contour lines indicate the

time-averaged angular footprint of embedded protein helices, enabling visualization of spatial correlations between protein location, membrane deformation, and lipid sorting. Angular coordinates are shown in degrees, with ϕ spanning 0–360° and θ spanning 0–180°. Color scales correspond to radial displacement or lipid enrichment, as indicated.

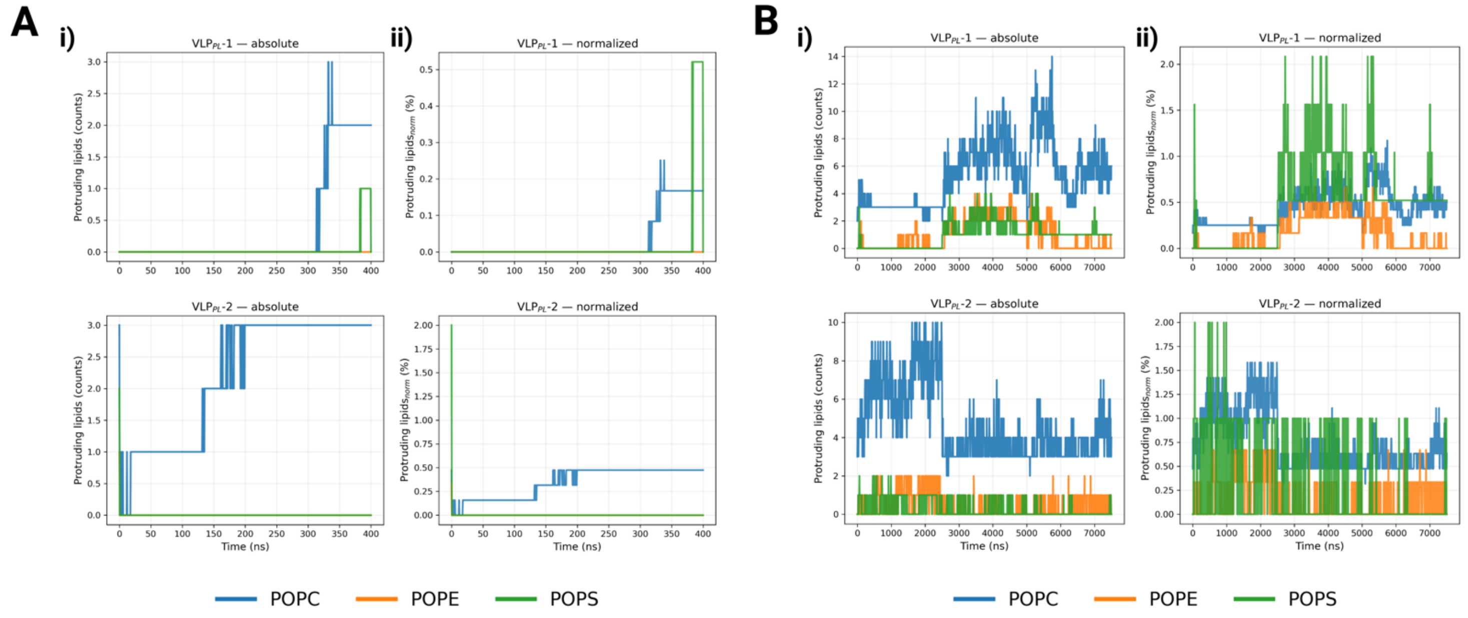

C

	POPC	POPE	POPS
VLP$_{PL}$-1 (mean count)	5.3 ± 2.3	1.0 ± 1.1	1.0 ± 0.9
VLP$_{PL}$-1 (normalized%)	0.444 ± 0.194	0.158 ± 0.180	0.502 ± 0.460
VLP$_{PL}$-2 (mean count)	4.7 ± 1.9	0.5 ± 0.6	0.3 ± 0.5
VLP$_{PL}$-2 (normalized%)	0.737 ± 0.294	0.163 ± 0.210	0.260 ± 0.461

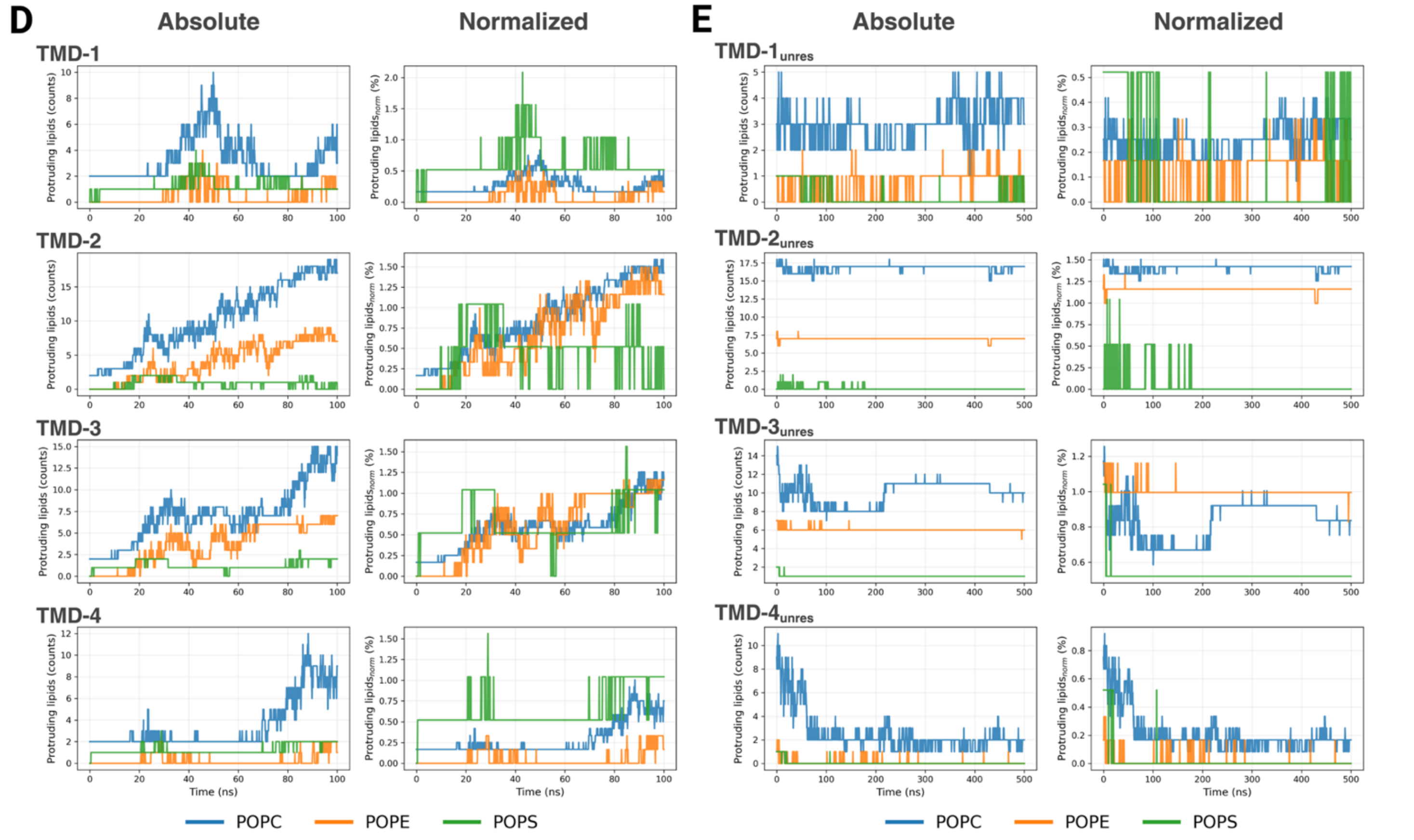

Figure S9. Time-resolved lipid protrusion and escape dynamics during coarse-grained molecular dynamics simulations of immature DENV-2 VLPs. (A–B) Time evolution of lipid protrusion or escape events from the lipid envelope during (**A**) equilibration simulations, in which protein backbone beads were restrained while lipids were free to rearrange (VLP_{PL}-1 and VLP_{PL}-2 of 400 ns each), and (**B**) unrestrained production simulations of VLP_{PL}-1 and VLP_{PL}-2 obtained from combined triplicate trajectories (3 × 2,500 ns). Plots report absolute counts (left panels) and composition-normalized escape fractions (right panels) for POPC, POPE, and POPS lipids. Lipid escape was defined using PO4 headgroup positions: at each frame, the vesicle center of mass was calculated from all lipid PO4 beads, the median radial distance was taken as the vesicle surface, and lipids with PO4 beads located more than 5.0 nm beyond this surface were classified as protruding or escaped. Absolute escape counts report the number of protruding or escaped lipid molecules over time, whereas normalized escape fractions represent the fraction of escaped lipids relative to the total number of each lipid species present in the vesicle, allowing direct comparison of escape propensity across lipid types despite differences in composition (POPC:POPE:POPS ≈ 6:3:1). **(C)** Summary table reporting the mean ± standard deviation of absolute and normalized lipid escape values for the unrestrained production simulations of VLP_{PL}-1 and VLP_{PL}-2, calculated from the combined triplicate trajectories. **(D–E)** Time-resolved lipid protrusion and escape during targeted molecular dynamics (TMD) simulations (**D**) and during subsequent 500 ns unrestrained CG-MD simulations initiated from the final frames of the TMD trajectories (**E**). Plots report absolute counts (left panels) and composition-normalized escape fractions (right panels) for POPC, POPE, and POPS lipids. During TMD, lipid protrusion events transiently increase in response to enforced protein rearrangement but remain limited in magnitude. Upon removal of biasing forces, protrusion frequencies stabilize or decrease, with no evidence of progressive accumulation or membrane destabilization, indicating that lipid excursions are reversible and not induced by the steering protocol.

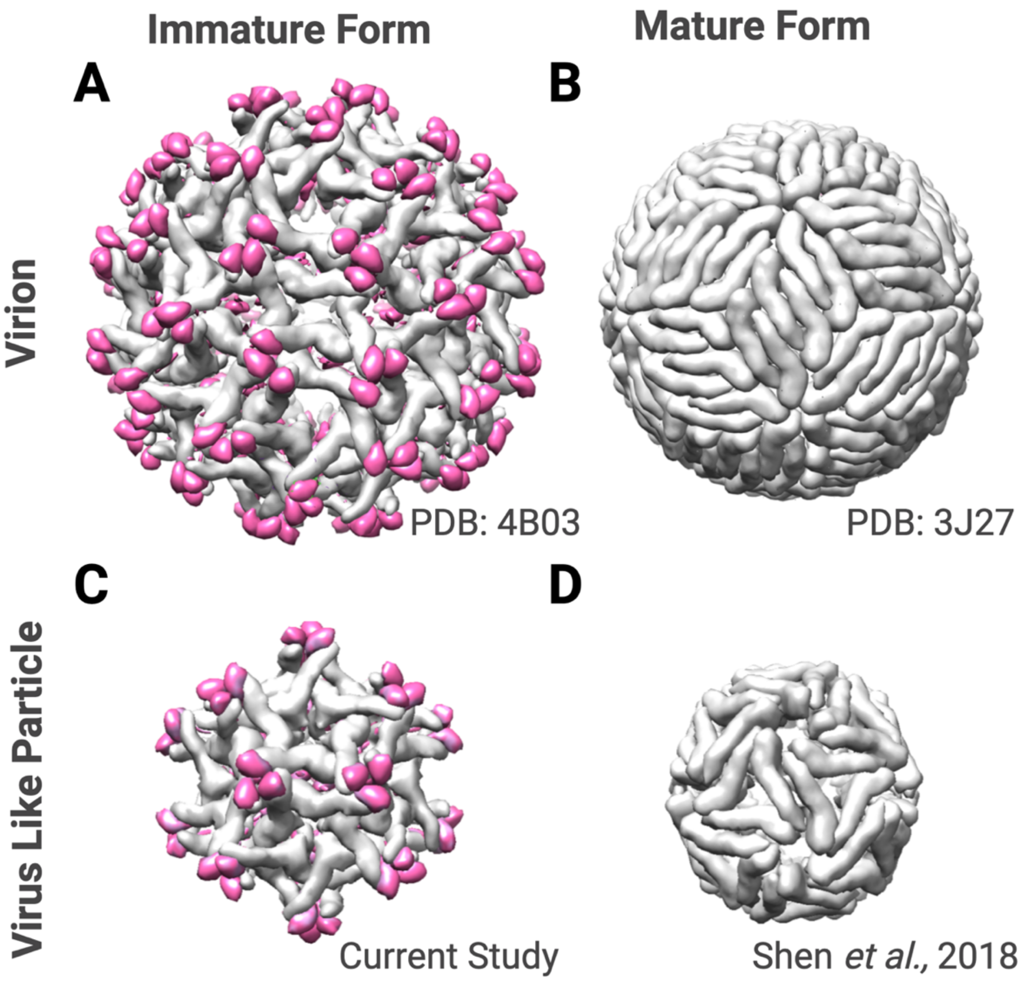

Figure S10. Structural arrangement of E and pr proteins in virion and VLP. Organization of the E and (pr)M proteins on the surface of **(A)** the immature virion, **(B)** the mature virion, **(C)** the immature VLP, and **(D)** the mature VLP is shown. Proteins are shown in surface representation: gray – E proteins, magenta – pr molecules.
